# Abstracts from the Fourteenth Rambam Research Day, December 7, 2017

**DOI:** 10.5041/RMMJ.10329

**Published:** 2018-01-29

**Authors:** Shraga Blazer, Ehud Klein

**Affiliations:** 1 Editor-in-Chief, Rambam Maimonides Medical Journal, Rambam Health Care Campus, Haifa, Israel; 2 Director, Division of Research, Rambam Health Care Campus, Haifa, Israel

Dear colleagues,

Maimonides, known as the *Rambam*, was one of the greatest Jewish arbiters; he was also a scientist, a researcher, and one of the greatest philosophers of the medieval ages. In fact, he was among the first to support evidence-based medicine.

This Supplement of *Rambam Maimonides Medical Journal* presents the abstracts from the Fourteenth Annual Rambam Research Day. These abstracts represent the newest basic and clinical research coming out of Rambam Health Care Campus—research that is the oxygen for education and development of tomorrow’s generation of physicians. Hence, the research presented on Rambam Research Day is the foundation for understanding patient needs and improving treatment modalities. Bringing research from the bench to the bedside and from the bedside to the community is at the heart of Maimonides’ scholarly and ethical legacy.

We hope you will appreciate the potential represented in these abstracts, which touch on nearly every aspect of clinical practice.

I would also like to thank Ms. Deborah Hemstreet and Ms. Lisbet Clements for their invaluable and professional editorial work that went into making this special issue of *Rambam Maimonides Medical Journal* possible.

*Rambam Maimonides Medical Journal* would be pleased to publish other original basic and clinical research articles, and related abstracts, which will subsequently be applied to patient care. In addition, we are open to publishing such abstracts from meetings, seminars, or conferences. For more information on seeing your event’s abstracts published here, please contact our editorial board.

Sincerely Yours,

Shraga Blazer, M.D. Editor-in-Chief On behalf of the Editors

