# Supplementary material for: Abstracts from the Fourteenth Rambam Research Day, December 7, 2017
Source: Rambam Maimonides Med J. 2018 Jan 29;9(1):e0010. doi: 10.5041/RMMJ.10329 (PMC5796741; doi:10.5041/RMMJ.10329)
Supplement: Related texts

# Abstract: About Rambam Research Day

**Published:** 2018-01

We are proud to introduce you to the Fourteenth Annual Rambam Research Day, now established as a key annual event at Rambam Health Care Campus (Rambam), reflecting the diverse research activities on our campus.

Integral to Rambam’s research activities are several competitive research funding programs that Rambam administers: The Horizons (*Ofakim)* program, which provides start-up and bridge research funding for young staff physicians with strong research credentials and plans, and the Leaders (*Atidim*) program, which is directed towards research training support for our residents. The Leaders program continues to receive wide recognition and serves as an especially important example of Rambam’s dedication to medical trainees and the development of physician scientists.

Two additional competitive research grant programs were recently initiated. In 2015 the “Guardian” (*MAOF*) program for Rambam’s nursing staff was initiated. Grants of up to NIS 10,000 are awarded to support research and innovative projects of the nursing staff. The “Foundations” (*MALKAM*) research program, initiated in 2016, offers up to NIS 20,000 for resident-initiated clinical trials. Both of these programs will encourage and facilitate research initiatives from new sectors of the hospital staff and further contribute to the advancement of medical practice at Rambam. This year we saw a new program added for the benefit of our clinical laboratory researchers—the “Optimum” (*METAV*) Program.

Combined, these research programs are critical components of personal professional development for Rambam’s medical personnel.

We would like to acknowledge the excellent collaboration and the fruitful research ties between Rambam Health Care Campus and the Technion–Israel Institute of Technology, in particular The Ruth & Bruce Rappaport Faculty of Medicine. We would also like to thank Professor Chaim (Howard) Cedar from the Hebrew University of Jerusalem as the Chair of our Scientific Advisory Board.

We are proud of the scientific abstracts that were presented at the meeting, and which compose this Supplement to *Rambam Maimonides Medical Journal*. The abstracts cover all areas of medicine. They reflect the rich spectrum of clinical, applied, and basic research endeavors that characterize our medical center’s activities. Indeed, our primary mission of bringing health care to the community is achieved as we combine these research discoveries with excellence in teaching.

We would also like to recognize the dedicated staff of Rambam’s Research Division, including the Institutional Review Board (IRB), and the Office for Advancement of Research; together they are responsible for the coordination and oversight of all research activities at Rambam, including clinical research, applied research, competitive grant management, patent protection, institutional review board ethical approvals, and bringing forward innovative ideas to clinical practice. We value collaboration with industry, which serves as an integral part of health care progress; over the years, several Rambam-originated research projects have led to remarkable success in Israel, with practical application to health care.

We are particularly pleased to see this Supplement to *Rambam Maimonides Medical Journal*, which has now become a tradition at the *Rambam Maimonides Medical Journal*, for the awareness and promotion of research activities in Israel, particularly at Rambam. Supporting Rambam Research Day via *Rambam Maimonides Medical Journal* is another way we show commitment to our patients, via the furthering of health care facilitated by progress in science, innovation, and technology.

**Rafi Beyar, M.D., D.Sc.** *Director, Rambam Health Care Campus and Associate Editor, Rambam Maimonides Medical Journal***Yehuda Chowers, M.D.** *Director, Clinical Research Institute at Rambam, Rambam Health Care Campus***Karl Skorecki, M.D.** *Director, Medical & Research Development, Rambam Health Care Campus and Associate Editor, Rambam Maimonides Medical Journal***Ehud Klein, M.D.** *Director, Division of Research, Rambam Health Care Campus*

# Abstract: Rambam Health Care Campus Research Day Organizing Committee

**Published:** 2018-01

**Professor Ehud Klein**

Director, Research Division

**Professor Shraga Blazer**

Editor-in-Chief, Rambam Maimonides Medical Journal

**Professor Benjamin Brenner**

Director, Department of Hematology and Bone Marrow Transplantation

**Professor Myriam Weyl Ben-Arush**

Director, Division of Pediatric Medicine, Ruth Rappaport Children’s Hospital Director, Division of Pediatric Hematology-Oncology and Bone Marrow Transplantation

**Professor Doron Norman**

Chair, Division of Surgery

**Professor Zeev Weiner**

Director, Department of Obstetrics and Gynecology

**Dr. Yael Shahor**

Deputy Director, Pediatric Division, Ruth Rappaport Children’s Hospital Attending Physician, Infectious Disease

**Assistant Professor Ron Beloosesky**

Director, Obstetrics and Gynecology Ultrasound Unit, Department of Obstetrics and Gynecology

**Dr. Gal Akiri**

Head of Research and Grants Management, Division of Research

**Mrs. Gila Hyams**

Director of Nursing; Nursing Director RHCC and Coordinator Nurse, Trauma and Emergency Center

**Mrs. Mirit Barzilai**

Director of Nursing, Operating Rooms

**Professor Marcia C. Javitt**

Director, Department of Medical Imaging

**Assistant Professor Yeshayahu (Shai) Katz**

Director, Department of Anesthesiology

**Dr. Rostislav Novak**

Orthopedic Surgery Section

**Dr. Eyal Fruchter**

Director, Division of Mental Health

**Assistant Professor Marielle Kaplan**

Director, Department of Laboratory Medicine

**Clinical Associate Professor Amir Karban**

Director, Department of Internal Medicine C

**Dr. Aeyal Raz**

Department of Anesthesiology

**Assistant Professor Sarig Galit**

Director, Hematology Laboratory

# Abstract: Program for the Fourteenth Rambam Research Day

**Published:** 2018-01

**Thursday, December 7, 2017**

Rambam Health Care Campus, Haifa, Israel

07.00 **Meet & Greet**

**Moderator: Professor Ehud Klein**

Director, Research Division, Rambam Health Care Campus

07.30 **Opening Session: Greetings**

**Professor Rafi Beyar**

Director, Rambam Health Care Campus

**Professor Shimon Marom**

Dean, The Ruth & Bruce Rappaport Faculty of Medicine, Technion–Israel Institute of Technology (IIT), Haifa, Israel

**Thanksandgreetings to Professor Klein upon his retirement from his position as the director of the Research Division**

**Professor Karl Skorecki**

Director, Medical & Research Development, Rambam Health Care Campus

**Professor Yehuda Chowers**

Director, Clinical Research Institute at Rambam (CRIR), Rambam Health Care Campus

**Honorary Lecturer**

07.50 **Professor Eran Elinav**

Eran Elinav, M.D., Ph.D. | HHMI & the Bill and Melinda Gates Foundation International Research Scholar | Senior Fellow, Canadian Institute for Advanced Research (CIFAR) | Principal Investigator, Host–Microbiome Interaction Research Group | Weizmann Institute of Science

**Host–Microbiome Interactions in Health and Disease**

09.00 **Announcementof Internal Rambam Research Grant Recipients**

09.20 **Best Rambam Research Day Posters Awards**

*10.00* ***Oral Presentationsin Two Simultaneous Sessions***

# Abstract: Choosing Wisely: Mechanical Ventilation in Older Patients in Internal Medicine Departments. (Poster #38)

**Published:** 2018-01

**Authors:** Tzvi Dwolatzky, Bella Smolin, Leon Levi, Ayelet Raz-Pasteur, Tatiana Mashiach, Daniel King

**Affiliations:** 1 Geriatric Unit, Rambam Health Care Campus, Haifa, Israel; 2 Internal Medicine Unit, Rambam Health Care Campus, Haifa, Israel; 3 Administration, Rambam Health Care Campus, Haifa, Israel; 4 Epidemiology and Statistics Unit, Rambam Health Care Campus, Haifa, Israel

## Background

The aging of the population is associated with increased morbidity. Technological developments have made an important impact on promoting longevity. Mechanical ventilation is widely available and used for respiratory support, and this has resulted in increasing numbers of older ventilated patients in Internal Medicine Departments, placing greater demands on the health system both in acute and chronic care.

## Study Aims

The aim of this study was to determine the factors involved in the decision-making process leading to mechanical ventilation of older people, and to evaluate the results of mechanical ventilation in patients treated in Internal Medicine Departments.

## Materials & Methods

We performed a prospective observational study over a period of 18 months. All patients aged 65 years and older who received respiratory support by means of mechanical ventilation and who were treated in one of the five Internal Medicine Departments or in the Medical Intensive Care Unit at Rambam Health Care Campus were included. Demographic and medical data were collected as well as details regarding the decision-making process and outcomes.

## Results

Our study population included 619 patients, 568 of whom were ventilated for the first time, 25 were chronically ventilated, and 26 were reventilated. Mean age was 78.5 years, 42% were in their eighth decade, 32% in their ninth decade, and 6.8% were 90 years and older. There were 299 (48%) women and 320 (52%) men. Outcome was poor, with 60.6% in-hospital mortality. For those who survived the hospitalization, 18.7% were discharged home, 20.7% were transferred to nursing homes, and 21% required chronic ventilation in specialized units. Of those who were weaned from ventilators, 14.8% remained with permanent tracheostomy tubes. Only 12% achieved the “best outcome,” defined as successful weaning during hospitalization, discharge to their own home, and survival at 6 months post-discharge.

## Conclusions

Mechanical ventilation is initiated for respiratory support even at advanced old age. Poor survival and outcome emphasize the need to re-evaluate the use of mechanical ventilation as a life-saving measure in patients at the end of life. In addition, we believe that the policy regarding the use of mechanical ventilation in older patients with life-threatening conditions by paramedics in the community should be carefully re-evaluated.

# Abstract: Isolated Mitochondria Transplantation Restores Schizophrenia-related Deficits. (Poster #33)*In Vitro* and *In Vivo*

**Published:** 2018-01

**Authors:** Hila Ene, Odile Robicsek, Rachel Karry, Rotem Ben-Yehuda, Ofer Yitzhaki, Ina Wiener, Dorit Ben-Shachar

**Affiliations:** 1 Research Laboratory of Psychobiology, Department of Psychiatry, Rambam Health Care Campus, Haifa, Israel; 2 The Ruth & Bruce Rappaport Faculty of Medicine, Technion–Israel Institute of Technology, Haifa, Israel; 3 Laboratory of Behavioral Psychobiology, Department of Psychology, School of Psychological Sciences, Tel Aviv University, Tel Aviv, Israel

## Background

Schizophrenia (SZ) is a severe mental disorder with a worldwide prevalence of 1%. Malfunction of mitochondria, key players in various essential cell processes, has been recurrently reported in the disorder. Recently, induced pluripotent stem cells (iPSCs) derived from hair follicle keratinocytes of schizophrenia patients have shown a severely reduced ability to differentiate into dopaminergic neurons and inability to mature into glutamatergic neurons. Both of the two neurotransmitter systems are altered in the disorder. More recently, functional improvement of mitochondrial function has been observed in several disease models following mitochondrial transplantation.

## Study Aims

Here we aimed to study the effect of transplantation of isolated active normal mitochondria (IAN-MIT) on mitochondrial function, neuronal differentiation, and schizophrenia-related behavioral responses. This was studied in two experimental models: (1) schizophrenia-derived iPSCs differentiated into glutamatergic neurons; and (2) polyinosinic-polycytidilic acid [poly(I:C)] maternal immune activation rat model of the disorder. Notably, mitochondrial dysfunction has been observed in these two experimental models.

## Materials & Methods

In the *in vitro* study, IAN-MIT were transferred to SZ-derived iPSCs differentiating into glutamatergic neurons. At the end of the differentiation period, the efficiency of neuronal differentiation was assessed by the expression of neuronal and glutamatergic markers and glutamate and glutamine metabolism. In the *in vivo* study, pregnant dams were injected with poly(I:C) or saline. In adolescence, their offspring were administered with a single bilateral stereotaxic injection of freshly prepared IAN-MIT or vehicle into the medial prefrontal cortex. In adulthood, attentional function was assessed by the latent inhibition (LI) test, and mitochondrial function was evaluated in fronto-cortical freshly isolated JC-1 stained neurons by measuring mitochondrial membrane potential (ΔΨm) and mitochondrial network dynamics.

## Results

Transfer of IAN-MIT resulted in improved differentiation of schizophrenia-derived iPSCs into neurons and their glutamatergic activity. In rats, a prolonged effect on schizophrenia-related behavior and mitochondrial function in adulthood was demonstrated following cerebral injections of IAN-MIT in adolescence. In adult poly(I:C)-exposed offspring, IAN-MIT prevented disrupted LI and restored their reduced mitochondrial ΔΨm. In control offspring, however, an opposite pattern was observed.

## Conclusions

These findings demonstrate a beneficial long-lasting effect of IAN-MIT transplantation *in vitro* and *in vivo*, suggesting its therapeutic potential in diseases with bioenergetic and neurodevelopmental abnormalities such as schizophrenia. The results suggest that *in vivo* IAN-MIT transplantation is beneficial when mitochondrial function is impaired, while under normal conditions it interferes with the homeostasis of mitochondrial diverse functions. Moreover, this study provides evidence for a possible relationship between mitochondria and attention.

# Abstract: Modulation of Cancer Cell Metabolism by Macrophages. (Poster #40)

**Published:** 2018-01

**Authors:** Eran Fridman, Yoav Binenbaum, Neta Milman, Tomer Shlomi, Ziv Gil

**Affiliations:** 1 Department of Otolaryngology Head and Neck Surgery, Rambam Health Care Campus, Haifa, Israel; 2 The Ruth & Bruce Rappaport Faculty of Medicine, Technion–Israel Institute of Technology, Haifa, Israel; 3 The Clinical Research Institute at Rambam Clinical Research Institute at Rambam (CRIR), Rambam Health Care Campus, Haifa, Israel; 4 Department of Computer Science, Technion–Israel Institute of Technology, Haifa, Israel; 5 Department of Biology, Technion–Israel Institute of Technology, Haifa, Israel

## Background

Reprogramming cell metabolism is a central phenomenon in cancer and considered one of the hallmarks of cancer. Altered metabolic networks give cancer cells a proliferative advantage but also present an opportunity to develop new treatment strategies. Recent evidence suggests that the cancer microenvironment (specifically, cancer-associated fibroblasts) reprograms cancer cell metabolism and contributes to cancer progression. The metabolic effects exerted by the immune cells infiltrating the cancer microenvironment on cancer cells have not been investigated.

Exosomes are nanosized vesicles containing various molecular constituents of their cell of origin, including proteins and nucleic acid material. Exosomes play a role in many biological processes, including cancer biology. We have recently shown that tumor-associated macrophages (TAM) secrete exosomes that are shuttled to cancer cells conferring chemotherapy resistance.

## Study Aims

The aim of this study was to evaluate the metabolic reprogramming of TAM-secreted exosomes induced in cancer cells.

## Materials & Methods

We analyzed gene expression in cancer cells co-cultured with TAM exosomes compared to non-treated cancer cells. Next we functionally evaluated the effect of exosomes on cancer cells in terms of proliferation and extra-cellular acidification rate as a measure of glycolysis.

## Results

Co-culturing exosomes with cancer cells increased the expression levels of mRNA transcripts in the glycolysis and pentose phosphate pathway. Functionally, TAM exosomes increased both proliferation and glycolysis rate.

## Conclusions

Our results indicate a key role for cancer metabolic reprogramming by TAM via exosomes, highlighting the therapeutic potential of targeting the immune system.

# Abstract: Transcutaneous Electrical Functional Stimulation for Treatment of Premature Ejaculation: Results of a Proof of Concept Study. (Poster #71)

**Published:** 2018-01

**Authors:** Ilan Gruenwald, Boaz Appel, Omar Massarwi, Ravit Yehieli-Cohen, Arik Shechter

**Affiliations:** Neurology Unit, Rambam Health Care Campus, Haifa, Israel

## Background

Some 16%–38% of men of all ages experience premature ejaculation (PE), resulting in a significantly lower quality of sexual life. Transcutaneous electrical functional stimulation (TEFS) has been found to be effective in activating superficial musculature and therefore could theoretically inhibit the emergence of the bulbocavernous phasic contractions at the beginning of the ejaculatory process, thus delaying PE.

## Study Aims

The aim of this study was to evaluate the application of TEFS at the perineal region and its effect on prolonging time to ejaculation.

## Materials & Methods

In a previous study on 19 healthy males we were able to demonstrate that TEFS at the perineal region is safe and does not result in any discomfort, pain, or tissue damage. The procedure involves placing two small dermal patches on the perineum and connecting the patches to a stimulator.

This study included 23 men aged 18–60 with self-reported PE. Each patient participated in the study on two separate days. To familiarize the patients with the sensory stimulation and to identify individual sensory and motor thresholds, each patient underwent a demonstrative TEFS to the perineum during both visits, and was asked to indicate the maximal painless and comfortable stimulatory threshold. Patients were then randomly assigned to undergo either TEFS or sham the first week, followed by the alternating treatment a week later. Each patient was placed in a private, adequately set-up room, where the treatment or sham was initiated. Each patient was asked to self-stimulate in privacy, while measuring the time from full erection to ejaculation using a stopwatch. Feasibility of retarding ejaculation was determined by analysis of differences in measured time to ejaculation between both groups (TEFS vs control [Sham]).

## Results

Of the 23 men included in the study, two dropped out due to difficulty achieving erections, and one was lost to follow-up. Twenty men aged 22–54 (mean 37.5 years) completed the study. In 16 patients, time to ejaculation was increased, and the overall average increase was more than twofold (2:04 min in the sham group vs 5:11 min in the TEFS group, *P*=0.00057). Receiving TEFS in the first or the second visit did not affect the results. No side effects were reported.

## Conclusions

This study demonstrates for the first time that TEFS is a feasible treatment modality that can significantly prolong the time to ejaculation. Further research on a larger group of patients in the home setting, with a partner, and using a wireless device has already been approved.

# Abstract: Elucidating the Novel Interaction Between mTOR and Glycolysis Towards Discovering Therapeutic Targets Based on Cancer-specific Pathways. (Poster #13)

**Published:** 2018-01

**Authors:** Lena Ilan, Nevraj Kejiou, Stefan Aigner, Enching Luo, Ines Rabano, Nishani Rajakulendran, Eeliza Lee, Eric Van Nostrand, Stephane Angers, Gene Yeo, Gilad Amiel, Alexander Palazzo

**Affiliations:** 1 Department of Urology, Rambam Health Care Campus, Haifa, Israel; 2 Department of Biochemistry, University of Toronto, Toronto, Ontario, Canada; 3 Institute for Genomic Medicine, University of California San Diego, San Diego, CA, USA; 4 Leslie Dan Faculty of Pharmacy, University of Toronto, Toronto, Ontario, Canada

## Background

For successful growth and propagation, cancer cells acquire a number of properties, including the ability to grow independently of external growth signals and accumulating glycolytic intermediates as building blocks for macromolecules such as DNA, RNA, proteins, and lipids. Many malignancies select for mutations that allow permanent activation of pathways promoting growth and propagation. The mechanistic target of the rapamycin (mTOR) pathway and glycolysis are among the most important pathways altered in most cancer types.

Under unfavorable conditions, mTOR pathway inhibition causes downregulation of protein synthesis. The most responsive to mTOR inhibition are mRNAs participating in ribosome biogenesis that contain cis-elements called 5′ terminal oligo pyrimidine track (5′TOP). In cancer cells, aberrant permanent activation of mTOR causes constitutive protein synthesis. Additionally, mTOR activation promotes glycolysis upregulation and causes accumulation of glycolytic intermediates to enable uncontrolled growth and propagation of the cancer cell. Our preliminary results suggest involvement of glycolytic enzymes in 5′TOP mRNA regulation.

## Study Aims

The aim of this study was to identify the mechanisms of ribosome biogenesis regulation by glycolytic enzymes.

## Materials & Methods

We used human osteosarcoma cell line (U2OS) to isolate cytoplasmic polysomes, mRNA-protein complexes that actively synthetize new proteins. Protein composition of the polysomes was analyzed by means of mass spectrometry. To analyze mRNAs that interact with the major glycolytic enzyme PKM, we immunoprecipitated tagged protein and performed enhanced UV crosslinking and immunoprecipitation (eCLIP). To evaluate if PKM affects mRNA translation, we tethered recombinant PKM to luciferase reporter construct.

## Results

Our findings show that most if not all glycolytic enzymes bind cytoplasmic polysomes. Further analysis of the mRNAs that co-immunoprecipitated with the major glycolytic enzyme PKM shows strong enrichment of a subset of mRNAs containing 5′TOP elements. Tethering of recombinant PKM to reporter luciferase construct resulted in the construct translation attenuation.

## Conclusions

Glycolysis up-regulation by mTOR activation is well described in the literature. We discovered a novel interaction between these two pathways with 5′TOP mRNAs directly regulated not only by mTOR pathway, but also by glycolysis. 5′TOP mRNAs encode proteins of ribosome biogenesis that constitutes a core process in cancer cell growth. Further understanding of the whole diversity of the interactions between these two major regulatory pathways, mTOR and glycolysis, will allow us effectively to inhibit cancer-specific protein synthesis and thus impair cancer cell viability.

# Abstract: Highly Effective Heparanase-based Therapy for Non-small Cell Lung Cancer. (Poster #37)

**Published:** 2018-01

**Authors:** Amit Katz, Uri Barash, Yaniv Zohar, Ilanit Bovango, Neta Ilan, Ran Kremer, Israel Vlodavsky

**Affiliations:** 1 Department of General Thoracic Surgery, Rambam Health Care Campus, Haifa, Israel; 2 Cancer and Vascular Biology Research Center, The Ruth & Bruce Rappaport Faculty of Medicine, Technion–Israel Institute of Technology, Haifa, Israel; 3 Department of Pathology, Rambam Health Care Campus, Haifa, Israel

## Background

Heparanase is an enzyme capable of cleaving heparan sulfate (HS) side chains of heparan sulfate proteoglycans. Heparan sulfate cleavage results in remodeling of the extracellular matrix (ECM) and is associated with enhanced tumor metastasis. Heparanase expression is augmented in numerous cancers. Previous studies have shown that heparanase levels are substantially increased in non-small cell lung cancer (NSCLC), correlating with reduced patient survival.

## Study Aims

The aim of this study was to reveal the significance of heparanase in the pathogenesis of lung cancer.

## Materials & Methods

We employed human (A549, HCC827 HTB182) and mouse (Lewis lung carcinoma) lung cancer cell lines to examine cellular invasion, colony formation in soft agar, and tumor growth following heparanase overexpression or inhibition. We further established a patient-derived xenograft (PDX) model system to examine the efficacy of heparanase inhibitor, PG545.

## Results

Heparanase overexpression augmented cellular invasion through reconstituted ECM, it increased colony formation in soft agar assay, and it enhanced tumor growth more than 2-fold vs. control (*P*<0.004). Moreover, cellular invasion, colony formation, and tumor growth were markedly attenuated by the heparanase inhibitor PG545 (*P*<0.005). Mechanistically, we found reduced Akt phosphorylation levels in cells treated with PG545, whereas the expression of p27, an inhibitor of the cell cycle, was increased.

In order to bring anti-heparanase treatment closer to the clinic, we established a PDX model of lung cancer. Fifteen patients with lung cancer (8 adenocarcinoma, 7 squamous cell carcinoma) were enrolled and signed an informed consent for using a biopsy of their tumor for research. Sixty percent of the biopsies (9/15) developed tumor xenograft that was sufficiently large to be advanced into an experiment. The tumor biopsies exhibited typical high heparanase activity that was maintained in the corresponding PDX. Remarkably, PG545 treatment resulted in reduced tumor growth in 89% of the PDXs. We found that PG545 treatments also inhibited macroscopic tumor metastasis. In this regard, heparanase inhibition is unique in its ability to restrain the growth of the primary lesion and its resulting metastases. Moreover, we showed that PG545 is highly effective in PDX (i.e. a patient) that was unresponsive to conventional chemotherapy (cisplatin), thus nicely demonstrating the concept and need for personalized medicine.

## Conclusions

High levels of heparanase resulted in a more aggressive tumor phenotype. Heparanase inhibitors (i.e. PG545) effectively restrained the growth and metastasis of lung cancer PDXs. More research is required to bring anti-heparanase treatment closer to the clinic.

# Abstract: A *CHEK2* Mutation Causes Six Different Neoplastic Tumors and Multiple Somatic Chromosomal Translocations in a Single Individual. (Poster #23)

**Published:** 2018-01

**Authors:** Nitzan Sharon Shwartzman, Elizabeth Half, Adi Mory, Lina Basel Salmon, Tamar Paperna, Alina Kurolap, Nivin Moustafa Hawash, Ahuvit Bassous, Christina Srouji, Hagit Baris Feldman

**Affiliations:** 1 The Genetics Institute, Rambam Health Care Campus, Haifa, Israel; 2 Technion–Israel Institute of Technology, Haifa, Israel; 3 Gastroenterology Institute, Rambam Health Care Campus, Haifa, Israel; 4 Recanati Genetics Institute, Beilinson Hospital, Rabin Medical Center, Petah Tikva, Israel; 5 Sackler Faculty of Medicine, Tel Aviv University, Tel Aviv, Israel; 6 Pediatric Genetics Unit, Schneider Children’s Medical Center of Israel, Petah Tikva, Israel; 7 Felenstein Medical Research Center, Rabin Medical Center, Petah Tikva, Israel

## Background

A 67-year-old male patient, the offspring of a consanguineous marriage, was initially referred to the gastrointestinal cancer prevention service due to polyposis since age 35 years. His cancer history was positive for thymoma at age 45 years, breast cancer at 65 years, and prostate cancer at 66 years. At evaluation, renal cell carcinoma (RCC) was observed on CT, and he was referred for nephrectomy and subtotal colectomy for multiple polyps. The surgical specimen from his colon consisted of ~50 tubular adenomas (polyps) and a 35-mm sigmoid gastrointestinal stromal tumor.

## Study Aims

The aim of this study was to identify the underlying genetic cause in a high-risk, seemingly Mendelian, cancer patient by using next-generation sequencing (NGS) technologies.

## Materials & Methods

Genetic analysis utilized whole-exome sequencing with in-house bioinformatic analysis. A karyotype analysis of white blood cells (WBCs) from the patient was also performed due to a single report on an occurrence of familial thymoma with a constitutional translocation. Follow-up karyotyping of bone marrow, fibroblasts, and RCC tissue was performed.

## Results

A homozygous missense mutation, p.Gly167Arg, was identified in *CHEK2*. Karyotype analysis of the patient’s WBCs revealed different multiple chromosomal translocations involving various chromosomes, in 40%–60% of the cells (observed independently in three tests by two cytogenetics laboratories). The genomic instability phenotype is somatic and evolving, as translocations were not found in the patient’s bone marrow, fibroblasts, and RCC.

## Conclusions

Here we describe a homozygous missense mutation in *CHEK2* suggested as the cause of adult-onset multi-organ tumorigenesis and multiple somatic chromosomal translocations. Several studies have reported associations of germline mutations in *CHEK2* with increased susceptibility to breast and prostate cancer; recently, *CHEK2* mutations have also been detected among lung, thyroid, and young colorectal cancer patients. We are currently investigating whether the homozygosity for p.Gly167Arg abrogates the activity of the *CHEK2* protein, and underlies the translocation phenotype, which may explain the high-risk phenotype observed in this patient.

# Abstract: Limited Sampling Strategies for Therapeutic Drug Monitoring of Intravenous Busulfan in Children, Based on Linear Multiple Regression Modeling. (Poster #16)

**Published:** 2018-01

**Authors:** Zvi Teitelbaum, Laila Nassar, Inna Scherb, Dorit Fink, Yael Lurie, Norberto Krivoy, Yedidia Bentur, Edna Efrati, Daniel Kurnik

**Affiliations:** 1 Clinical Pharmacology and Toxicology Section, Rambam Health Care Campus, Haifa, Israel; 2 The Ruth & Bruce Rappaport Faculty of Medicine, Technion–Israel Institute of Technology, Haifa, Israel; 3 Laboratory for Clinical Toxicology, Pharmacology, Clinical Pharmacology and Toxicology Section, Rambam Health Care Campus, Haifa, Israel

## Background

Intravenous busulfan (BU), often used in conditioning for hematopoietic stem cell transplantation, has a narrow therapeutic index and highly variable pharmacokinetics (PK), requiring therapeutic drug monitoring (TDM). The area under the BU plasma concentration–time curve (AUC) is a measure of exposure. Non-optimal exposures may result in graft failure, disease relapse, or toxicity. Intense sampling (ISS) of nine samples over a 6-hour dosing interval is currently used for AUC estimation, but it is labor-intensive and expensive.

## Study Aims

The aim of this study was to develop reliable limited sampling strategies (LSS), based on four to five samples, for TDM of busulfan.

## Materials & Methods

Pharmacokinetic and demographic data of children treated with intravenous BU were extracted from our laboratory databases. Seventeen LSSs (each based on ~260 TDM records) were devised. For each LSS, multiple linear regression (MLR) on randomly selected sub-cohorts that included 50% of the records was used to calculate MLR coefficients that yielded AUC_LSS_ best fitting AUC_ISS_. To minimize selection bias, the randomization and the MLR procedure were repeated 1,000 times for each LSS. Mean MLR coefficients obtained in such repeated randomizations were used to calculate individual AUC_LSS_MLR_ for all the records in each LSS cohort. The agreement between these and AUC_ISS_ was estimated by Bland–Altman analysis. Using the ratio of recommended dose adjustments based on AUC_ISS_ or AUC_LSS_ (*R* = ), we estimated for each LSS the mean of *R*, 95% CI limits of the distribution of *R* values, and their % deviation (*DEV*) from mean *R*. The agreement between LSS and ISS was deemed satisfactory when |*DEV*|<12.5%, a criterion stricter than those used in published reports of development of LSS for BU.

## Results

Seven of the 17 LSSs (five based on four samples and two based on five samples) exhibited low bias and |*DEV*|<12.5%; i.e. less than 5% of the dose adjustment recommendations based on these LSSs deviated more than 12.5% from those based on ISS. All seven LSSs performed equally well independent of patient age.

## Conclusions

Any one of these seven LSSs can be recommended for clinical use with Israeli patients. The advantages of LSS (reduction of patient discomfort, ~50% reduction of nursing staff workload and blood loss, ~25% reduction of laboratory workload) may encourage wider utilization of BU TDM, allowing personalized dosing for enhanced safety and efficacy. Their adoption to other patient populations requires additional evaluation.

# Abstract: Identification of Electrocardiographic Characteristics in Silent Carriers of Mutant KCNQ1 Gene. (Poster #41)

**Published:** 2018-01

**Authors:** Samer Abu-Salem, Wisam Darawsha, Mahmoud Suleiman, Monther Boulous, Lior Gepstein, Miry Blich

**Affiliations:** 1 The Ruth & Bruce Rappaport Faculty of Medicine, Technion–Israel Institute of Technology, Haifa, Israel; 2 Department of Cardiology, Rambam Health Care Campus, Haifa, Israel

## Background

Congenital long-QT syndrome (LQTS) is an inherited channelopathy characterized by a prolonged corrected QT interval and is associated with an increased predisposition for polymorphic ventricular arrhythmias and sudden cardiac death (SCD) in young subjects. About 10%–40% of family members are silent mutation carriers but still exhibit a significant increase in the risk of SCD compared with unaffected family members. Clinical identification of silent carriers is challenging.

## Study Aims

The aim of this study was to identify electrocardiographic characteristics using exercise test and Holter monitoring of silent carriers of mutant KCNQ1 gene.

## Materials & Methods

We compared QT length and shortening and T wave morphology during electrocardiogram (ECG), exercise test, and 12-lead Holter monitoring in 14 silent carriers of KCNQ1 mutation with 14 healthy subjects.

## Results

When compared to a healthy control group, heterozygous silent carriers of mutant KCNQ1 gene presented inadequate ability of QT interval adaptation in response to rapid changes in heart rates (increase of 10 beats per minute in less than 60 s). We found a median QT shortening of 12.3 ms in the carriers versus 15.5 ms in the control group, *P*=0.013. The value of the area under the T wave divided by T wave amplitude at a constant heart rate was significantly higher in the carrier group compared to our control group (86.7 versus 75.9, *P*=0.04).

In multivariate analysis, QT shortening of less than 14.3 ms while the heart rate increases by 10 beats in less than 60 s was a risk factor in silent carriers (HR 9.9, CI 1.1–71.1, *P*=0.035).

## Conclusions

Abnormal QT shortening in response to rapid changes in heart rate, and increase in area under the T wave divided by T wave amplitude, may be useful tools for detecting LQTS1 concealed carriers.

# Abstract: Melanoma Self-identification: Can a Computerized System Replace the Physician?. (Poster #59)

**Published:** 2018-01

**Authors:** Gal Avineri, Oren Talmor, Emily Avitan-Hersh, Netanel Ratner

**Affiliations:** 1 Faculty of Electrical Engineering, Technion–Israel Institute of Technology, Haifa, Israel; 2 Department of Dermatology, Rambam Health Care Campus, Haifa, Israel; 3 The Ruth & Bruce Rappaport Faculty of Medicine, Technion–Israel Institute of Technology, Haifa, Israel; 4 Rafael Advanced Defense Systems, Haifa, Israel

## Background

Melanoma is the deadliest of skin cancers and is the main cause of skin cancer-related deaths. However, when it is detected in its early stages, the prognosis significantly improves. Novel deep learning methods and accessible smart-phone photography provide an opportunity to improve skin self-examination by physicians and patients, and to reduce melanoma mortality.

## Study Aims

The aims of this study were to examine the capability of a computerized system to visually diagnose melanoma, and to create a larger database of benign and malignant pigmented lesions.

## Materials & Methods

To address these goals, we trained a deep convolutional neural network on the International Skin Imaging Collaboration (ISIC) Archive dataset of melanoma and benign lesion images. Several different network architectures were considered showing promising results. Since deep learning methods highly depend on large amounts of training data, we created a website that serves as a platform for uploading labeled images, which are automatically being trained upon.

## Results

With the dataset mentioned above, using Google’s Inception-v3 methodology, the system achieves high scores in diagnosing melanoma (sensitivity and accuracy values of 84.8% and 84.5%, respectively). These values are comparable to published accuracy of dermatologists.

## Conclusions

Computerized systems may have an accuracy level similar to dermatologists in diagnosing melanoma. However, for the system to become applicable, larger cohorts of higher-quality, clinically annotated skin lesion imaging should be collected.

# Abstract: The Israeli Multi-Center Registry of Medical Cannabis (MC) for Chronic Pain: Current Findings. (Poster #49)

**Published:** 2018-01

**Authors:** Joshua Aviram, Dorit Pud, Bareket Schiff-Keren, Miriam Ogintz, Simon Vulfsons, Tamar Yashar, Haim-Moshe Adahan, SilviuBrill, Howard Amital, Itay Goor-Aryeh, Dror Robinson, Leslie Green, Refael Segal, Yacov Fogelman, Oren Tsvieli, Ben Yellin, David Meiri, Elon Eisenberg

**Affiliations:** 1 Faculty of Social Welfare and Health Sciences, University of Haifa, Haifa, Israel; 2 Rambam Health Care Campus, Haifa, Israel; 3 Schiff-Keren Pain Clinic, Tel-Aviv, Israel; 4 Institute of Pain Medicine, Rambam Health Care Campus, Haifa, Israel; 5 Yashar Pain Clinics, Tel-Aviv, Israel; 6 Pain Rehabilitation Unit, Chaim Sheba Medical Center, Tel Hashomer, Israel; 7 Institute for Pain Medicine, Sourasky Medical Center, Tel Aviv, Israel; 8 Internal Ward B, Chaim Sheba Medical Center, Tel Hashomer, Israel; 9 Institute for Pain Medicine, Chaim Sheba Medical Center, Tel Hashomer, Israel; 10 Orthopedic Research Department, Hasharon Hospital, Rabin Medical Center; 11 Meuhedet Health Services, Geriatric Medicine Center “Shmuel Harofeh,” Beer Yaacov, Israel; 12 Long Term Care Department, Geriatric Medicine Center “Shmuel Harofeh,” Beer Yaacov, Israel; 13 Pain clinic Leumit HMO, Beer Sheva, Israel; 14 Shoulder and Elbow surgery unit, Mayanei HaYeshua Medical Center, Bnei Brak, Israel; 15 Faculty of Biology, Technion–Israel Institute of Technology, Haifa, Israel; 16 The Bruce Rappaport Faculty of Medicine, Technion–Israel Institute of Technology, Haifa, Israel

## Background

Although the use of medical cannabis (MC) for chronic pain is rising, scientific evidence on its long-term effectiveness and safety is scarce.

## Study Aims

The aim of this study was to set up a patient registry on the treatment of MC for the relief of chronic pain in Israel, by prospectively collecting data on the effectiveness and safety of MC.

## Materials & Methods

Patients with chronic pain for whom an application for using MC was completed were requested to complete a set of web-based questionnaires at baseline and 1, 3, 6, 9, and 12 months following initiation of MC use. Data included demographics, pain diagnoses and treatment, concomitant medical conditions, pain intensity, pain disability index, pain catastrophizing scale, Pittsburgh sleep quality index, general anxiety disorder, Beck depression inventory, quality of life, and morphine equivalent dose. Hierarchical linear and logistic models were used to assess efficacy and adverse effects (AEs), respectively.

## Results

Baseline data on 1205 patients (674 men; age 49±16 years) showed: 52% were employed/ students. Neuropathic pain was the most common diagnosis (71%). Data on 676, 604, 428, 319, and 202 patients were available at the five follow-up time points, respectively. Average MC dose increased over time (20, 21, 25, 26, and 28 g, respectively). MC was consumed mostly by inhalation (85%) or as oil extracts (15%). A significant (*P*<0.0001) improvement was noted in all parameters over time. A 30% improvement from baseline in all parameters at all follow-ups was noted in 16%–67% of patients. Nearly 43% of patients reported AEs, mostly mild and transient. During the one-year treatment, the percentage of patients reporting AEs gradually decreased to 30%. Furthermore, some patients had reported various AEs. Fatigue and dizziness were the most frequent AEs, followed by increased appetite, dry mouth, and sweet cravings.

## Conclusions

The current findings suggest a moderate long-term improvement of chronic pain, associated symptoms, and outcome measures with MC treatment. In general, only a small number of patients reported AEs following MC treatment, a number which seems to decrease gradually over one year of treatment. As the study sample is heterogeneous, continuation of the follow-up is needed in order to increase the number of patients completing the one-year treatment, to understand reasons for withdrawal from treatment, identify predictors of treatment success or failure, and substantiate our current findings.

# Abstract: Mycosis Fungoides Associated with Anti TNFα Therapy. (Poster #58)

**Published:** 2018-01

**Authors:** Emily Avitan-Hersh, Michal Ramon, Reuven Bergman

**Affiliations:** 1 Department of Dermatology, Rambam Health Care Campus, Haifa, Israel; 2 The Ruth & Bruce Rappaport Faculty of Medicine, Technion–Israel Institute of Technology, Haifa, Israel

## Background

Indolent T cell lymphomas, including mycosis fungoides (MF), have been reported in patients undergoing anti-TNFα therapy.

## Study Aims

The aim of this study was to elucidate and characterize MF following anti-TNFα therapy.

## Materials & Methods

The clinical files of patients in whom MF appeared during anti-TNFα therapy in our department were studied.

## Results

Mycosis fungoides appeared in 6 patients undergoing anti-TNFα therapy. Patients were treated with adalimumab (n=5) and golimumab (n=1). Underlying diseases were psoriasis (n=2), arthritis (n=2), Crohn’s disease (n=1), and Hidradenitis suppurativa (n=1). Latency to MF development was 3–12 months (average 7 months). Four patients had folliculotropic MF, and two had patch/plaque MF. The T cell receptor gene rearrangement was monoclonal in three of six patients. The MF disappeared in four patients after discontinuation of therapy, and in an additional one patient following phototherapy. Re-challenge in one patient resulted in reappearance of MF, whereas ustekinumab replaced anti-TNFα in two patients without recurrence.

## Conclusions

Anti-TNFα therapy may induce MF, and cessation of therapy with/without phototherapy is usually associated with regression.

# Abstract: Endoscopic and Histologic Findings Correlate with Free Infliximab Found in Uninflamed Tissue in Inflammatory Bowel Disease Patients. (Poster #68)

**Published:** 2018-01

**Authors:** Haggai Bar-Yoseph, Alexandra Blatt, Sigal Pressman, Shiran Gerassy, Shomron Ben Horin, Yehuda Chowers

**Affiliations:** 1 Department of Gastroenterology, Rambam Health Care Campus, Haifa, Israel; 2 The Ruth & Bruce Rappaport Faculty of Medicine, Technion–Israel Institute of Technology, Haifa, Israel; 3 Department of Gastroenterology, Chaim Sheba Medical Center, Rambam Gan, Israel; 4 Sackler School of Medicine, Tel Aviv University, Tel Aviv, Israel

## Background

Anti-TNF agents are widely used in the treatment of inflammatory bowel diseases (IBD). Despite the fact that the intestine is the main therapeutic target, little or no information is available regarding the ratios of free and TNF-bound infliximab in the intestinal tissue.

## Study Aims

The aim of this study was to assess the presence of free versus TNF-bound infliximab in the intestinal tissue of IBD patients and its possible association with clinical outcomes.

## Materials & Methods

Protein was extracted from frozen intestinal tissues of infliximab-treated patients. Free infliximab and TNF-bound infliximab were detected using ELISA and normalized to tissue protein concentration. Concurrent serum drug levels (SDL), anti-drug antibodies (ADA), serum TNF-bound infliximab levels, patient’s pharmacotherapy, clinical response based on physician global assessment, endoscopic appearance (severity determined according to Mayo scoring in ulcerative colitis and endoscopist’s assessment of ulceration severity, extent of disease, and affected area in Crohn’s disease), and pathological results (severity determined by observing pathologist graded as normal, mild, moderate, and severe disease) at the time of colonoscopy were determined.

## Results

Twenty-four biopsies from 13 patients (11 Crohn’s disease and 2 ulcerative colitis patients) were tested. Non-inflamed tissue infliximab levels, but not inflamed tissue levels, correlated with SDL (*R*=0.8499, *P*=0.0037, false discovery rate = 0.0185) and were negatively correlated with the endoscopic appearance (*R*=−0.7214, *P*=0.0185) and pathological severity (*R*=−0.7959, *P*=0.0059). TNF-bound infliximab was measured in both inflamed and non-inflamed specimens and did not correlate with drug levels in the serum or tissue. Anti-drug antibodies were only detected in a single patient, precluding statistical analysis. Notably, no TNF-bound infliximab was measured in the serum.

## Conclusions

These findings show that pharmacokinetic–pharmacodynamic interaction, as measured by SDL, better reflects drug levels in healthy mucosa rather than inflamed mucosa, and suggests a more complex drug–target interaction in inflamed tissue, which cannot be explained by target binding only. Future studies assessing SDL dynamic changes during the process of mucosal healing may allow their use as surrogate markers for this purpose.

# Abstract: N-acetyl-cysteine Treatment Decreases Offspring Brain Injury Associated with Necrotizing Enterocolitis in a Rodent Model. (Poster #47)

**Published:** 2018-01

**Authors:** Ron Beloosesky, Osnat Zmora, Ola Sonik, Shraga Blazer, Yuval Ginsberg, Khatib Nizar, Zeev Weiner

**Affiliations:** 1 Department of Obstetrics and Gynecology, Rambam Health Care Campus, Haifa, Israel; 2 The Ruth & Bruce Rappaport Faculty of Medicine, Technion–Israel Institute of Technology, Haifa, Israel; 3 Department of Pediatric Surgery, Assaf Harofeh Medical Center, Tzrifin, Israel; 4 Sackler Faculty of Medicine, Tel Aviv University, Tel Aviv, Israel; 5 Department of Neonatology, Rambam Health Care Campus, Haifa, Israel

## Background

Preterm birth is associated with both brain injury and necrotizing enterocolitis (NEC). N-acetyl-cysteine (NAC) is a known anti-inflammatory and antioxidant agent.

## Study Aims

The aim of this study is to determine whether NAC treatment during pregnancy and/or in the neonatal period can attenuate brain injury associated with intestinal injury in a rat model of NEC.

## Materials & Methods

Newborn Sprague–Dawley rats were randomized into three groups: control (*n*=10), who were breast-fed and kept in room air; NEC (*n*=10) who were subjected to hypoxia (5% O_2_ for 10 minutes) and formula feeding three times daily; NEC-NAC (*n*=10) who received NAC (300 mg/kg intraperitoneally once daily) in addition to hypoxia and formula feeding. Two additional groups included pups of dams who were treated once daily with NAC (300 mg/kg intravenously) for the last 3 days of pregnancy; after birth, the pups were randomized into NAC-NEC (*n*=10), with hypoxia and formula feeding alone, or NAC-NEC-NAC (*n*=10), with additional postnatal NAC treatment. All pups were sacrificed on the fifth day, and the brains were harvested for protein studies. NFkB p65, nNOS, caspase3, TNFα and IL-6 protein levels were determined by western blot and compared between the groups by ANOVA. *P*<0.05 was considered statistically significant.

## Results

The NEC pups had significantly increased brain levels of NFkB p65, nNOS, caspase3, TNFα, and IL-6 compared to controls (0.26±0.01 vs 0.21±0.01 μ; 0.25±0.01 vs 0.21±0 μ; 0.21±0.01 vs 0.09±0.01 μ; 0.28±0.01 vs 0.22±0.01 μ; 0.29±0 vs 0.22±0 μ, respectively, *P*<0.05). In all NAC treatment groups the levels of NFkB p65, nNOS, caspase3, TNFα, IL-6, and IL-1β were significantly reduced compared to the NEC group. The most pronounced decrease was demonstrated within the NAC-NEC-NAC group (0.21±0.01 vs 0.26±0.01 μ; 0.21±0 vs 0.25±0.01 μ; 0.09±0.01 vs 0.21±0.01 μ; 0.22±0 vs 0.28±0.01 μ; 0.22±0 vs 0.29±0.0 μ, respectively, *P*<0.05).

## Conclusions

N-acetyl-cysteine treatment can decrease offspring brain inflammation associated with NEC in a rat model by inhibition of the NFkB, nNOS, and caspase3 pathways. Starting preventive treatment in pregnant patients at high risk for preterm labor might be a novel approach to decrease the risk or severity of brain injury in the newborn.

# Abstract: Fetal/Neonatal Alloimmune Thrombocytopenia: An Underestimated Life-threatening Clinical Condition. (Poster #54)

**Published:** 2018-01

**Authors:** Lilach Bonstein, Roy Lauterbach, Nardeen Atweh, Nuhad Haddad

**Affiliations:** 1 Platelet and Neutrophil Immunology Laboratory, Rambam Health Care Campus, Haifa, Israel; 2 Division of Obstetrics and Gynecology, Rambam Health Care Campus, Haifa, Israel; 3 Hematology and Bone Marrow Transplantation Institute, Rambam Health Care Campus, Haifa, Israel

## Background

In fetal/neonatal alloimmune thrombocytopenia (FNAIT), platelets (PLT) are destroyed by maternal antibodies directed against fetal antigens. Thrombocytopenia may be severe and lead to intracranial hemorrhage in about 10% of cases. FNAIT is estimated to be markedly underdiagnosed, partly due to the fact that complete blood count (CBC) is not routinely done in all neonates.

## Study Aims

The aim of this study was to evaluate the degree of awareness of FNAIT in an attempt to decrease the risk of this devastating condition.

## Materials & Methods

A retrospective analysis was performed of 322 suspected FNAIT cases sent to our reference laboratory over the past 4 years from the majority of medical centers in Israel.

## Results

The retrospective analysis revealed a low referral rate of 39%. In addition, 50% of the families with a thrombocytopenic newborn were referred for evaluation more than 4 weeks after discharge, when the antibody titer could already be below the detection level. Twenty-seven percent of the mothers with a previously suspected FNAIT pregnancy were referred for antibody evaluation only during a subsequent pregnancy. A supplementary retrospective analysis performed at our center between 2010 and 2015 showed that a CBC test was done for various clinical reasons only in 7,370 (23.1%) of 31,952 newborns. Thrombocytopenia (<150×10^9^ PLT/L) was found in 2,505 (34%) of these babies, being severe (<50×10^9^ PLT/L) in 220 (2.98%) of them. Extended evaluation of the latter subgroup demonstrated that 45 newborns were term and had no other possible causes for the low PLT count. Yet, only 7 babies were referred for FNAIT assessment: 4 of them were found positive for PLT antibodies. Thus, in the current analysis, 38/45 (84%) of the term newborns with severe thrombocytopenia were not assessed for FNAIT.

## Conclusions

Physician awareness and the cooperation of involved multidisciplinary teams (neonatologists, pediatric hematologists, and gynecologists) are crucial for FNAIT diagnosis. Development of uniform guidelines for the evaluation of this life-threatening clinical condition is warranted.

# Abstract: Evidence of Dietary Carrageenan as Cause of Intestinal Epithelial Stress, and Interference with Protein Digestion in Infants, Adults, and Elderly. (Poster #05)

**Published:** 2018-01

**Authors:** Shlomit David, Lulu Fahoum, Carmit Shani Levi, Ron Shaoul, Uri Lesmes, Ester G. Meyron-Holtz, Geila S. Rozen

**Affiliations:** 1 Faculty of Biotechnology and Food Engineering, Technion–Israel Institute of Technology, Haifa, Israel; 2 Department of Nutrition, Rambam Health Care Campus, Haifa, Israel; 3 Department of Pediatric Gastroenterology, Rambam Health Care Campus, Haifa, Israel

## Background

Carrageenan (CGN) is a marine polysaccharide, approved as a food additive (E407). Three commercial CGN types (termed κ-, ι-, and λ-CGN) are widely used in processed foods as thickeners and stabilizers. Various studies have raised concerns about CGN safety due to indications suggesting a causal connection between CGN and human gastrointestinal health. Evidence implies that CGN may interfere with digestive proteolysis. Partial digestion consequently impairs the physiologic intestinal homeostasis including the intestinal epithelium function, thus questioning the safety of CGN use in foods.

## Study Aims

The aim of this study was to understand functional and morphological impacts of digested CGN in models of different human populations.

## Materials & Methods

The three industrial CGNs were subjected to a semi-dynamic *in vitro* digestion system mimicking human digestion in healthy infant, adult, or elderly populations. Gastric and duodenal proteolysis of whey protein isolate (WPI) under gastrointestinal (GI) conditions were examined. Digesta were also incubated with an intestinal epithelium model, the human colon carcinoma cell line Caco-2, in order to elucidate effects on intestinal epithelial function and their potential role in the progression of inflammatory bowel diseases (IBD).

## Results

Carrageenans were found to significantly interfere with enzymatic activity of pepsin gastric proteolytic enzyme. It also affected WPI digestion in the infant, adult, and elderly GI *in vitro* model.

Overnight incubation of confluent Caco-2 monolayers with physiologically digested CGNs (pd-CGNs) altered gut permeability, cell morphology, and protein distribution in tight junctions (TJs). Furthermore, pd-CGNs induced expression of the inflammation marker CXCR1.

## Conclusions

All CGNs hindered WPI gastro-intestinal proteolysis in adults and elderly and affected WPI proteolysis to varying extents in infants under *in vitro* GI conditions. Pd-CGN clearly caused morphological and functional disruption of the intestinal epithelial model.

We hypothesize that digestion changes the chemical composition of CGN, consequently subjecting the GI tract to deleterious unsuspected effects. Mechanistic aspects and impacts of sumption on pre-existing inflammation are currently being investigated.

# Abstract: Regulation of CXCR2 Receptor Signaling in Tumor-infiltrating T-cells by Lnk Adaptor Protein. (Poster #31)

**Published:** 2018-01

**Authors:** Yael Derdikman, Yasmine Khier, Shuny Peled, Ella Kenigswald, Michal Hayun, Igal Louria-Hayon, Yishai Ofran

**Affiliations:** 1 The Ruth & Bruce Rappaport Faculty of Medicine, Technion–Israel Institute of Technology, Haifa, Israel; 2 The Clinical Research Institute at Rambam (CRIR), Rambam Health Care Campus, Haifa, Israel; 3 Hematology and Bone Marrow Transplantation Institute Rambam Health Care Campus, Haifa, Israel; 4 The Faculty of Biology, Technion–Israel Institute of Technology, Haifa, Israel

## Background

The adaptor protein Lnk is mainly and vastly expressed throughout the hematopoietic system. Through its SH2 domain, Lnk is able to bind a large variety of phosphorylated tyrosine residues on intracellular protein targets. The binding of Lnk to an activated target protein of a cytokine receptor pathway leads to an inhibition of its downstream signaling cascade. The regulatory balance between bound and non-bound Lnk enables regulation of the cellular response to cytokine stimulation.

In humans, an increasing number of Lnk mutations have been demonstrated in myeloproliferative neoplasm (MPN) patients, leading to an increased cellular proliferation, probably due to high activity of the Jak-STAT pathway. Lnk-deficient (Lnk^−/−^) mice exhibit an immune-mediated intestinal inflammation and an MPN-like phenotype, which presents with an enlarged spleen and abnormal blood count.

## Study Aims

The aim of this study was to examine the role of Lnk regulation in the tumor immune-microenvironment.

## Materials & Methods

Lnk^−/−^ mice were used as a model in our study. B16-F10 melanoma cells were injected subcutaneously to Lnk^−/−^ and wild-type (WT) mice, and tumors were harvested after 14 days. Tumors were dissolved into a single-cell suspension. Single cells were analyzed using flow cytometry, and tumor proximal fluids were analyzed for cytokine composition using ELISA. CD8^+^ and CD4^+^ T-cells were separated from the heterogenic splenocytes population by negative selection. Phosphorylation of signaling proteins was examined by western blot.

## Results

Tumors of Lnk^−/−^ mice were one-third the size of tumors of WT mice, and presented an increased population of tumor-infiltrating lymphocytes (TILs).

An extensive analysis of the immune subpopulations at the tumor microenvironment pointed out an increased population of tumor-infiltrating cytotoxic CD8^+^ T-cells (CTLs).

Analysis of the cytokine profile at the tumor proximal fluids revealed an increased presence of the murine IL-8 analog Mip-2, which binds the CXCR2 chemokine receptor.

*Ex vivo* stimulation of spleen-derived Lnk^−/−^ CD8^+^ T-cells and CD4^+^ T-cells with Mip-2 resulted in an enhanced phosphorylation of STAT3 and Erk1/2.

## Conclusions

Our findings suggest that Lnk may be a novel regulator of CXCR2 signaling. This implies an involvement of Lnk regulation in the tumor microenvironment, and may position Lnk as a target for immunotherapy.

# Abstract: Anti-TNF Therapy Reverses B-cell Senescence in Inflammatory Joint Disease. (Poster #45)

**Published:** 2018-01

**Authors:** Garrett Friedman, Doron Melamed, Doron Markovits, Yonit Tavor, Firas Sabbah, Alexander Rozin, Kohava Toledano, Katya Dolnikov, Naomi Finkilshtein, Yolanda Braun-Moskovici, Alexandra Balbir-Gurman

**Affiliations:** 1 The Ruth & Bruce Rappaport Faculty of Medicine, Technion–Israel Institute of Technology, Haifa, Israel; 2 Melamed Laboratory for Developmental Immunology and Aging, The Ruth & Bruce Rappaport Faculty of Medicine, Technion–Israel Institute of Technology, Haifa, Israel; 3 B. Shine Rheumatology Unit Rambam Health Care Campus, Haifa, Israel

## Background

Tumor necrosis factor (TNF)-α is a key molecule in the pathway of systemic inflammation. Inflammatory joint diseases (IJD), such as rheumatoid arthritis, psoriatic arthropathy, and spondyloarthropathy, are associated with high circulating levels of TNF-α. TNF-α has a suppressive effect on B-cell production in the bone marrow and promotes early B-cell senescence, resulting in reduced immune function, increased morbidity, and poor response to vaccination. In mice TNF-α therapy has a rejuvenating effect on B-cell population, increased lymphopoiesis, and peripheral diversity of the B-cells. Transitional B-cells represent a population of newly generated cells that migrate from the bone marrow to complete their selection and maturation in the periphery; quantification of these cells in peripheral blood reflects ongoing B lymphopoiesis.

## Study Aims

We hypothesize that in patients with IJD active inflammation imposes senescence of the B-cell compartment and impairs immune function. We postulate that anti-TNF-α therapy in such patients not only reduces inflammation activity but also replenishes the senescent B-cell population, leading to a reversal of immune compromise, and better outcomes.

## Materials & Methods

Immunofluorescence staining for flow cytometric analysis was performed on previously incubated peripheral blood mononuclear cells; B-cells were identified both morphologically and by positive CD19 expression. Transitional B-cells were further identified as CD24-positive and CD38-positive.

The study population included healthy controls (HC, *n*=6), IJD on non-biological treatment (IJD-NB, *n*=4), and IJD before (active phase) and three months after starting anti-TNF-α therapy (IJD-TNF, *n*=7).

## Results

In a cross-sectional analysis, the transitional B-cell compartment of IJD patients was reduced to a third compared to that of HC (*P*<0.01). We further observed that patients with IJD treated with anti-TNF-α therapy had a 2.67-fold increase in the transitional B-cell compartment (*P*<0.01) compared to the IJD-NB group. In a longitudinal analysis of IJD-TNF patients, a three-month anti-TNF-α therapy was associated with a 4.66-fold increase in the transitional B-cell population (*P*<0.01, *n*=4).

## Conclusions

Chronic inflammation in patients with IJD suppresses B lymphopoiesis and promotes early B cell senescence. Anti-TNF-α therapy, in addition to controlling the inflammation, reactivates B lymphopoiesis, reverses B cell aging, and in this way could restore immune response to infection or vaccination. Expansion of this study to a large patient population is ongoing.

# Abstract: Patients’ Needs and Satisfaction during Emergency Room Visits. (Poster #72)

**Published:** 2018-01

**Authors:** Roni Gagin, Neta HaGani, Itay Levy, Aziz Darawsha, Hava Lutinger

**Affiliations:** 1 Department of Social Work, Rambam Health Care Campus, Haifa, Israel; 2 Department of Emergency and Urgent Care Medicine, Rambam Health Care Campus, Haifa, Israel

## Background

Medical staff treat hundreds of patients who arrive at the emergency room (ER) every day. The heavy ER patient load creates long waiting times and puts an emotional and physical burden on both patients and staff. Efforts are invested in reducing patient load and long waiting times in the ER. However, it is not clear how those efforts reflect in reality. It is important to learn from patients about their needs and satisfaction from services provided in the ER in order to improve services and allocation of resources.

## Study Aims

The aim of this study was to examine factors associated with satisfaction from treatment in the ER among patients, in order to improve services and patients’ experiences as part of patient-centered care.

## Materials & Methods

A cross-sectional survey was conducted among 114 patients who visited the ER of Rambam Medical Center. Patients were approached randomly while waiting in the ER and, upon agreement, were interviewed by a social worker. The questionnaire included questions regarding satisfaction from: ER physical conditions (organization, cleaning, etc.), waiting times, amount of information received, and communication with staff.

## Results

Over 50% reported a high general satisfaction from the ER visit; 35% of patients reported waiting in the ER for over 8 hours. The highest level of satisfaction was from communication with staff, and the lowest level of satisfaction was from the amount of information received and from the physical conditions of the ER. General satisfaction from the ER visit was positively associated with communication with staff, amount of information received, and ER physical conditions, and negatively associated with waiting times. Patients who reported receiving enough information also reported shorter waiting times, more positive communication with staff, and more organization in ER. It was also found that older patients experienced more positive communication with staff, shorter waiting times, and a higher general satisfaction from ER visit.

## Conclusions

Patients report a high general satisfaction from their ER visit, but were not satisfied with the amount of information they receive regarding their medical status and ER procedures. The physical conditions of the ER are also important for a patient’s satisfaction from the ER visit. Patients and their families should be included in the treatment plan. The hospital should consider improving ER physical conditions and the ways information is distributed to patients by using advanced technology, and by supporting the medical staff who work under constant pressure.

# Abstract: Prevention of Fetal Brain Injury: Progesterone Modulation of Inflammation. (Poster #46)

**Published:** 2018-01

**Authors:** Yuval Ginsberg, Ola Gutzeit, Ofer Fainaru, Nizar Khatib, Zeev Weiner, Ron Beloosesky

**Affiliations:** Department of Obstetrics & Gynecology, Rambam Health Care Campus, Haifa, Israel

## Background

Maternal progesterone (P) administration has been shown to reduce the risk of preterm birth in women at high risk for preterm delivery (PTD). OPPTIMUM (dOes Progesterone Prophylaxis To prevent preterm labour IMprove oUtcoMe?), the largest randomized trial of vaginal progesterone for prevention of preterm birth in women at high risk, found a reduction in neonatal brain injury evident by ultrasound in the progesterone (P) group.

## Study Aims

We sought to determine the neuroprotective effect of maternal progesterone treatment in a maternal inflammation model.

## Materials & Methods

Pregnant mice (ICR strain; *n*=24) were pretreatment randomized to vaginal P (1 mg/ day) or carrier (Replens) from day 13 to day 16 of gestation. At days 15 and 16, either lipopolysaccharide (LPS 15 μg) or vehicle was intraperitoneally administered. Mice were sacrificed 4 hours following last LPS/saline injection. Fetal brains were collected from the four treatment groups (6 in each group) (P/normal saline [SAL], P/LPS, Replens/LPS, Replens/SAL [control]). Levels of nuclear factor kappa-light-chain-enhancer of activated B cells (NFkB), neurol nitric oxide synthases (nNOS), interleukin 6 (IL6), and tumor necrosis factor α (TNFα) were determined by western blot.

## Results

Maternal LPS (Replens/LPS) significantly increased fetal brain levels of nNOS (0.24±0.03 vs. 0.08±0.01μ), NFkB (0.23±0.01 vs. 0.16±0.01 μ), IL6 (0.33±0.01 vs. 0.11±0.01 μ), and TNFα (0.3 ±0.02 vs. 0.09±0.01 μ), compared to control fetuses (*P*<0.01). Maternal progesterone supplementation significantly reduced fetal brain levels of nNOS (0.24±0.03 vs. 0.14±0.01 μ), NFkB (0.23±0.01 vs. 0.16±0.01 μ), IL6 (0.33±0.01 vs. 0.14±0.01 μ), and TNFα (0.3±0.02 vs. 0.2±0.06 μ) to levels similar to controls (Replens/SAL).

## Conclusions

Maternal progesterone for the prevention of PTD might protect the fetal brain from inflammatory-induced fetal brain injury by inhibition of specific inflammatory and oxidative pathways.

# Abstract: Contrast-induced Nephropathy: A Comparison of Two Agents. (Poster #65)

**Published:** 2018-01

**Authors:** Yuri Gorelik, Gali Levy, Hiba Yaseen, Mogher Khamaisi

**Affiliations:** 1 Department of Internal Medicine D, Rambam Health Care Campus, Haifa, Israel; 2 Clinical Research Institute at Rambam, Rambam Health Care Campus, Haifa, Israel; 3 The Ruth & Bruce Rappaport Faculty of Medicine, Technion–Israel Institute of Technology, Haifa, Israel

## Background

Nephropathy following the administration of iodine-based contrast agents (IBCA) is common and associated with increased morbidity and mortality. Early reports have suggested that gadolinium-based contrast agents (GBCA) for enhancement of magnetic resonance imaging (MRI) studies do not confer significant risk of nephrotoxicity, but later studies have shown that this risk is similar and possibly greater with GBCA than with IBCA. These studies were performed on relatively small groups of patients (ranges of about 20–200) and usually analyzed a modest number of variables.

## Study Aims

The aim of the current study was to assess the risk of nephrotoxicity of GBCA in comparison to IBCA. The secondary aim was to identify patient characteristics that might be associated with increased risk for IBCA- or GBCA-induced nephropathy.

## Materials & Methods

This was a retrospective single-center data analysis. We have extracted data on all adult inpatients who underwent a computerized tomography (CT) or a MRI imaging procedure. Propensity score matching and logistic multiple regression were used to compare the risks of nephropathy for different contrast agents and assess the effect of various patient characteristics on these risks.

## Results

Patients had undergone either GBCA-enhanced MRI (*n*=1,379) or IBCA-enhanced CT (*n*=17,040). Risk of nephropathy in matched pairs was significantly lower for GBCA (6.13% vs 8.91%, McNemar’s chi-square *P* value = 0.004). Multiple logistic regression factors such as age, chronic kidney disease, and low hemoglobin levels are significantly associated with nephropathy risk in GBCA and IBCA groups. Furthermore, diabetes and fever pre-imaging are associated with risk of IBCA nephropathy but not with GBCA nephropathy.

## Conclusions

Our findings suggest that GBCA confers an overall lower risk of nephropathy as compared to IBCA nephropathy especially in the presence of certain baseline characteristics.

# Abstract: The Safety and Efficacy of CO_2_ Laser Therapy for Female Stress Urinary Incontinence. (Poster #75)

**Published:** 2018-01

**Authors:** Ilan Gruenwald, Roy Lauterbach, Hanin Dabaja, Lior Lowenstein

**Affiliations:** 1 Neuro-Urology Unit, Rambam Health Care Campus, Haifa, Israel; 2 Department of Gynecology, Rambam Health Care Campus, Haifa, Israel

## Background

Female stress urinary incontinence (SI) is highly prevalent in females and has a significant negative effect on quality of life. The surgical treatment for SI tends towards less invasive procedures. Recently laser therapy has been introduced as a new non-invasive therapy for SI and is based on applying fractional photonic energy to the vaginal mucosa tissue, causing micro-ablation and rapid healing, mainly affecting collagen and strengthening the surrounding supporting tissue.

## Study Aims

The aim of this study was to evaluate the safety and efficacy of the CO_2_ laser for treatment of female SI.

## Materials & Methods

We included females aged 18–70 with demonstrated SI by medical history, physical examination, and urodynamic testing, with documented normal Pap smear. Pregnancy, recurrent urinary tract infections, and vaginal bleeding were exclusion criteria. Prior to laser therapy, each patient completed validated SI and prolapse questionnaires (UDI6 and ICIQ-UI). The patients were re-evaluated 1 and 3 months post-therapy by both physical examination and the aforementioned questionnaires. In addition all patients were re-evaluated 3 months post-therapy by urodynamic testing.

Laser therapy was performed without anesthesia, and each treatment session lasted up to 5 minutes. We used the US Food and Drug Administration (FDA) approved AcuPulse™ CO_2_ Laser with an adapted vaginal probe-FemTouch™ handpiece (Lumenis, Yokneam Industrial Park, Israel). Pulsed laser energy covered all the vaginal mucosa area. Each patient underwent three sessions, with a 4-week interval between each treatment. During each visit a full evaluation of safety and efficacy was performed using the same evaluation tools.

## Results

Ten of 17 screened women, mean age 53, completed the 3-month follow-up period. Of these, 40% were post-menopausal. The average number of births was 3.3. In two cases, at the end of the last visit, some discomfort (sense of too much heat) was reported that did not prevent completing the treatment. Overall, no significant side effects were reported during or after treatment. At 3 months, 50% had a significant clinical improvement and another 30% were dry. No change was seen in two females. The average score of the validated questionnaires showed significant improvement in incontinence and related complaints; an improvement in the ICIQ-UI incontinence questionnaire score by 5.3 points (*P*=0.0008); and an improvement by 17.2 in the UDI6 questionnaire (*P*=0.0009). In 40% of the females, urodynamics still demonstrated stress incontinence.

## Conclusions

CO_2_ Laser therapy for SI is safe and effective and could be considered a good therapeutic alternative, especially in females who failed physiotherapy and pelvic floor rehabilitation, for females who prefer a more conservative approach, or in potential high-risk cases for surgery.

# Abstract: Development of a Novel Discreet Device for the Non-invasive Evaluation of Bladder Outlet Obstruction: Initial Results on Applicability, Safety, and Accuracy. (Poster #74)

**Published:** 2018-01

**Authors:** Ilan Gruenwald, Omar Massarwi, Ravit Yehieli-Cohen, Boaz Appel

**Affiliations:** Neuro-Urology Unit, Rambam Health Care Campus, Haifa, Israel

## Background

Currently, the gold-standard method for distinction between obstructive and non-obstructive uropathy is the pressure/flow study (PFS) obtained from routine urodynamic studies, which is invasive and involves urethral catheterization and bladder irrigation, both risk factors for infection and discomfort. A search for a less invasive test that will yield the same information led to the development of the PeePal System (P-square Medical, Herzliya, Israel) which has been previously shown to correlate well only with standard flowmetry results.

## Study Aims

The aim of this study was to evaluate the applicability and safety of the non-invasive PeePal and to compare its recorded flow parameter results to the standard PFS data.

## Materials & Methods

We included men aged 18–85 who came to the neuro-urology unit for a urodynamic study. A disposable ring-like vibro-acoustic sensor connected to a portable recording device was placed externally on the dorsum of the penis. The input signals during flowmetry were recorded and analyzed, and their characteristics were identified (specifically various degrees of laminar and turbulent flow). Accordingly, we determined if the flow was unobstructed, equivocal, or obstructed. After completing the full urodynamic test, the recorded PeePal results were compared to those obtained from the PFS data.

## Results

In this ongoing trial, 28 recruited men underwent a full urodynamic test while wearing the sensor during flowmetry. In all 28 subjects, the sensor was easily applied, did not cause any discomfort, and did not interfere with the voiding process. Signal quality was good, and size or girth of the penis did not prevent applying the device in comfort. There were no safety issues. We excluded four samples that showed low signal-to-noise ratio due to poor skin sensor contact and as part of the development process; the remaining signals were used for calibration, modification of the algorithm, and fine-tuning of the recordings. At this stage, data from four recruited subjects were used for true comparison with the PFS data, showing an excellent 1:1 correlation between the final results (obstructive vs. non-obstructive flow) from both studies.

## Conclusions

These preliminary results show promise for a new non-invasive methodology that may replace, in the near future, the invasive standard PFS. This technology could also be used in the home setting. Further work is required for establishing the accuracy of the new device.

# Abstract: Safety and Feasibility of VL#FIA3-30, a New Topical Solution for Treating Erectile Dysfunction: A Preliminary Study. (Poster #76)

**Published:** 2018-01

**Authors:** Ilan Gruenwald, Omar Massarwi, Boaz Appel

**Affiliations:** Neuro-Urology Unit, Rambam Health Care Campus, Haifa, Israel

## Background

To date there is no effective topical therapy for erectile dysfunction (ED) because the tunica albuginea is impenetrable to various drugs. Local application of a dermal solution that can traverse the tunica is expected to be more effective and less associated with side effects.

## Study Aims

The aim of this study was to assess the feasibility and safety of VL#FIA3-30 (VasoLead Ltd, Tel Aviv, Israel), a new solution composed of a combination of moxisylyte and isosorbide di-nitrate (ISDN), two vasoactive substances immersed in a unique tunica-penetrable delivery system.

## Materials & Methods

We included men with a moderate ED score of 11–19 based on the International Index of Erectile Function, Erectile Function Domain (IIEF-EF). These patients were randomly divided into four groups. Pharmacokinetics were measured in Group 1; safety and feasibility were evaluated in Groups 2–4 (which differed in number of applications: 2, 3, and 4 times, respectively).

At the first visit, after a washout period lasting two weeks, all subjects filled out validated sexual function questionnaires and non-validated informational questions regarding the product’s effect. After local application, blood pressure (BP) levels were measured every half hour during the first 2 hours, followed by hourly BP measurement for the following 4 hours. For safety reasons, any drop in BP ≥20 mmHg was considered an exclusion criterion and the patient was considered a screen failure. Associated side effects (headache, weakness, dermal toxicity, discomfort, and irritation) were recorded. The IIEF-EF and Sexual Encounter Profile (SEP) were completed by each participant every visit.

## Results

Thirty-two ED patients with a mean IIEF-EF score of 14.5 were included. Three had a BP drop of ≥20 mmHg (without any clinical effect) and were excluded. Only 3 men reported side effects; two had transient irritation around the meatus, and one had a headache (with no change in BP).

In Group 1 (eight patients), after applying VL#FIA3-30, plasma levels for characterizing the pharmacokinetics of VL#FIA3-30 were measured every 15 minutes during the first hour, three times after the second hour, once after the third hour, and then at 6, 12, 24, and 36 hours after application. No significant concentrations of moxisylyte or ISDN nor their active metabolites (desacetyl-moxisylyte, isosorbide mononitrate [ISMN]-2, ISMN-5; plasma concentrations 10.2, 12.7, 15.0, ng/mL, respectively) were detected in the tested samples.

Clinically significant penile engorgement was reported in one-third of the patients. Six men were able to complete full intercourse (in combination with an ED medication), and two patients were able to do so without any medical support.

A significant increase in IIEF-EF score from 14.4 to 19.4 was seen only in Group 4 (4 applications, *n*=7). Of these, three reported a mean increase of 8 points.

## Conclusions

Our results show that applying VL#FIA3-30 in men with ED is feasible and safe. Studies on larger numbers can now be conducted to evaluate clinical efficacy of this solution.

# Abstract: Heparan Sulfate Chains Contribute to an Anticoagulant Milieu in Malignant Pleural Effusion. (Poster #06)

**Published:** 2018-01

**Authors:** Emilia Hardak, Eli Peled, Yonatan Crispel, Shourouk Ghanem, Judith Attias, Inna Kogan, Benjamin Brenner, Yona Nadir

**Affiliations:** 1 Pulmonology Institute, Rambam Health Care Campus, Haifa, Israel; 2 The Ruth & Bruce Rappaport Faculty of Medicine, Technion–Israel Institute of Technology, Haifa, Israel; 3 Division of Orthopedics, Rambam Health Care Campus, Haifa, Israel; 4 The Thrombosis and Hemostasis Unit, Rambam Health Care Campus, Haifa, Israel; 5 STAT Laboratory, Rambam Health Care Campus, Haifa, Israel

## Background

Malignant pleural effusion (MPE) is a common and significant cause of morbidity in cancer patients, although the utility of the present treatment options is limited. The coagulation system is a major contributing factor to tumor growth and angiogenesis. Human heparanase, known to be involved in angiogenesis and metastasis, cleaves heparan sulfate (HS) side chains on the cell surface and in the extracellular matrix, yielding HS fragments of still appreciable size (5–7 kDa).

## Study Aims

In the present study we explored the coagulation milieu in MPE and infection pleural effusion (IPE).

## Materials & Methods

A total of 30 MPE and 44 IPE were compared to 33 transudate pleural effusions using heparanase ELISA, heparanase procoagulant activity assay, thrombin and factor Xa chromogenic assays, and thromboelastography. Proliferation assay was used. A cell line of mouse breast cancer (EMT-6) was injected to the pleural cavity of mice. Peptide 7 that inhibits heparanase activity was injected subcutaneously.

## Results

Heparanase level, procoagulant activity, and levels of factor Xa and thrombin were significantly higher in exudate than in transudate. In thromboelastography with the MPE no thrombus was detected. This effect was reversed when the assay was done with bacterial heparinase that degrades HS chains to small chains that have no effect in the coagulation system. Proliferation of human glioma (U87) and human breast carcinoma (T47D) cell lines was higher with addition of exudate than transudate. Infection pleural effusion induced more proliferation than MPE (*P*<0.001), but this was reversed when bacterial heparinase was added to the malignant effusion. Tumors in the pleural cavity of mice treated with peptide 7 were significantly smaller compared to control (*P*<0.0001).

## Conclusions

Heparin sulfate chains, probably released by heparanase, create an anticoagulant milieu in MPE that prevents local thrombosis and fibrosis. Inhibition of heparanase may provide a therapeutic option in patients with recurrent MPE.

# Abstract: BDNF Val66Met and Serum Protein Levels are Associated with Perception and Development of Vincristine-induced Peripheral Neuropathy in Patients with Non-Hodgkin Lymphoma. (Poster #42)

**Published:** 2018-01

**Authors:** Netanel Horowitz, Roni Naser, Rivka Sharon, Liya Simanovich, Luiza Akria, Ety Shaoul, Andrei Braester, David Azoulay

**Affiliations:** 1 Institute of Hematology and Bone Marrow Transplantation, Rambam Health Care Campus, Haifa, Israel; 2 The Ruth & Bruce Rappaport Faculty of Medicine, Technion–Israel Institute of Technology, Haifa, Israel; 3 Department of Internal Medicine B, Rambam Health Care Campus; 4 Hematology Unit and Laboratories, Galilee Medical Center, Nahariya, Israel

## Background

Vincristine-induced peripheral neuropathy (VIPN) is a major neurological side effect and dose-limiting factor in patients with lymphoma. Brain-derived neurotrophic factor (BDNF) is a neuronal growth factor that is important for the maintenance and repair of the central and peripheral nervous system.

## Study Aims

The aim of this study was to evaluate the role of the common BDNF gene single nucleotide polymorphism (Val66Met-SNP) and peripheral blood BDNF protein levels in non-Hodgkin lymphoma (NHL) patients developing VIPN.

## Materials & Methods

Peripheral neuropathy was assessed and graded in 33 non-Hodgkin lymphoma patients at diagnosis, and at the middle and at the end of vincristine-based therapy, using the reduced version of Total Neuropathy Score (TNSr) and the Functional Assessment of Cancer Therapy/ Gynecologic Oncology Group-Neurotoxicity (FACT-GOG-NTx) questionnaire. The Patient Health Questionnaire (PHQ9) questionnaire was used to assess depression. BDNF protein level was quantified in patients’ serum using commercial ELISA kit, and Val66Met-SNP was determined by Sanger sequencing.

## Results

There was no genotype-dependent difference in baseline and maximal TNSr scores. Patients with only one polymorphism of the BDNF gene (Val-BDNF) showed a trend of higher baseline FACT-GOG-NTx score and significantly elevated maximal FACT-GOG-NTx and PHQ9 scores as compared to patients with a second polymorphism of the BDNF gene (Met-BDNF). A significant inverse correlation was revealed between baseline BDNF protein levels and maximal TNSr and FACT-GOG-NTx scores. No such correlation was demonstrated with the maximal PHQ9 scores (TNSr: *r*=0.612, *P*=0.001; FACT-GOG-NTx: *r*= 0.643, *P*=0.0007; and PHQ9: *r*=0.24, *P*=0.2). Reduced BDNF protein levels in the serum along the follow-up were observed in Met-BDNF patients and not in Val-BDNF patients.

## Conclusions

Our results suggest an association between Val-BDNF SNP and increased perception of VIPN. In both genotypes, higher baseline serum BDNF levels may be protective against VIPN development.

# Abstract: Psychotropic Drugs Demonstrate a Specific Mitochondrial Effect Profile: Implication for Personalized Medicine in Psychosis Disorders. (Poster #32)

**Published:** 2018-01

**Authors:** Wessal Hussien, Ofer Yitzhaki, Tamara Ifraimov, Odeya Damri, Yuly Bersudsky, Galila Agam, Dorit Ben-Shachar

**Affiliations:** 1 Research Laboratory of Psychobiology, Department of Psychiatry, Rambam Health Care Campus, Haifa, Israel; 2 The Ruth & Bruce Rappaport Faculty of Medicine, Technion–Israel Institute of Technology, Haifa, Israel; 3 Department of Clinical Biochemistry and Pharmacology and Psychiatry Research Unit, Faculty of Health Sciences, Ben-Gurion University of the Negev and Mental Health Center, Beer-Sheva, Israel; 4 Psychiatry Research Unit, Division of Psychiatry, Faculty of Health Sciences, Ben-Gurion University of the Negev and Mental Health Center, Beer-Sheva, Israel

## Background

Schizophrenia and bipolar disorder patients exhibit emotional cognitive and behavioral abnormalities. Their life-time world prevalence is about 1% and their onset is at late adulthood. Both disorders also share symptoms, genetic risks, and endophenotypes. Despite extensive research their etiology is still unknown, and their current pharmacological treatment is based on a “trial and error” strategy. Abnormalities in various functions of the mitochondria have been reported for both disorders; some of them are targeted by psychotropic drugs.

## Study Aim

The aim of the present study was to identify a psychotropic drug-induced mitochondrial profile as a means for personalized treatment for psychotic patients. We therefore studied the effect of psychotropic drugs on various mitochondrial functions in healthy subjects and psychotic patients derived lymphocytes.

## Materials & Methods

Patients diagnosed by DSM-V for schizophrenia and bipolar disorder (*n*=50) and healthy controls (*n*=36) were recruited following approval by the Ethical Committee of the Mental Health Center Beer-Sheva. Fresh isolated peripheral lymphocytes were treated with six different psychotropic drugs and assessed for the levels of 14 proteins involved in various mitochondrial processes; autophagy (Beclin1, p62), apoptosis (Caspase 3, BAX, BCL2), mitochondrial network dynamics (OPA1, MFN1, DRP1, FIS1) and the electron transport chain (NDUFV1, NDUFV2, NDUFS1, COX2, SDH1), by indirect ELISA, as well as mitochondrial cellular distribution, network connectivity and membrane potential (Δψm) by confocal microscopy.

## Results

In healthy subjects, *in vitro* treatment resulted in a drug-specific profile of the effect on the mitochondrial parameters. A similar effect of the drugs was observed in chronically treated patients (*in vivo*) and in a small sample size (*n*=6) of non-treated patients (*in vitro*). Notably, in responding, but not in non-responding patients, the *in vitro* (before treatment) and the *in vivo* (after treatment) response to given drugs showed a high and statistically significant correlation for about half of the parameters.

## Conclusions

Our findings provide proof of concept that mitochondrial function parameters are a potential tool to *a priori* predict an individual’s response to a particular psychotropic medication (personalized medicine) and encourage further studies in order to establish mitochondrial-based profiles to facilitate such predictions.

# Abstract: Grb2 Regulates Proliferation and Survival of Hematopoietic Stem Cells. (Poster #30)

**Published:** 2018-01

**Authors:** Yasmine Khier, Yael Derdikman, Michal Hayun, Yishai Ofran, Igal Louria-Hayon

**Affiliations:** 1 Technion–Israel Institute of Technology, Haifa, Israel; 2 The Clinical Research Institute at Rambam, Rambam Health Care Campus, Haifa, Israel; 3 Department of Hematology and Bone Marrow Transplantation, Rambam Health Care Campus, Haifa, Israel

## Background

Although hematopoietic stem cell (HSC) proliferation, survival, and expansion have been shown to be supported by the cooperative action of different cytokines, little is known about the intracellular signaling pathways that are activated by cytokines upon binding to their receptors. Growth factor receptor-bound protein 2 (Grb2) is a ubiquitous adaptor protein known to be involved in signaling induced by a variety of growth factors and cytokines; however, its physiological role in HSC has never been characterized.

## Study Aims

The aim of this study was to examine the physiological role of Grb2 protein in HSC.

## Materials & Methods

At first, to examine the expression of Grb2 in HSC, we analyzed Grb2 mRNA expression in different purified mouse cells of the hematopoietic hierarchy by using quantitative RT-PCR technique.

To test the physiological role of Grb2 in HSC, we used a conditional Grb2^fl/fl^ knockout (KO) mouse model that we bred to Mx1-Cre mice to obtain conditional deletion of Grb2 in hematopoietic cells by injection of polyinosinic-polycytidylic acid (poly(I:C)). All control (Grb2^fl/fl^ = WT) and mutant (Grb2^fl/fl^-Mx1-Cre = Grb2-KO) mice were treated with three injections of poly(I:C). To check the integrity of our system, we examined the expression of Grb2 in the bone marrow (BM) of both WT and Grb2-KO mice by western blot technique.

To evaluate the effect of Grb2 deletion on the regenerative capacity of HSC, we transplanted unfractionated bone marrow cells, collected from donor WT or Grb2-KO mice in competition with recipient cells, into lethally irradiated recipient mice. Eight weeks after transplantation, when injected cells had stably engrafted, we induced Grb2 deletion with four injections of poly(I:C) and analyzed donor-derived lineage reconstitution 4, 8, 16, and 24 weeks after Grb2 deletion using flow cytometry technique.

To examine the effect of Grb2 deletion on the regenerative capacity of HSC after chemotherapy treatment, Grb2-KO and WT mice were treated once with 150 mg/kg 5-fluorouracil (5-FU), and HSC cell cycle status was investigated via flow cytometry technique; in addition, hematopoietic recovery was analyzed by neutrophil blood counts 6 and 13 days after chemotherapy treatment. We also performed apoptosis assay in order to examine the survival of HSC after 5-FU treatment.

## Results

Our study has shown that Grb2 mRNAs are highly expressed in HSC relative to more differentiated cells of the myeloid and erythroid lineages. Conditional deletion of Grb2 induced a progressive decline of long-term (LT-) HSC numbers and cytopenia with animal aging. While Grb2 deletion did not modify LT-HSC quiescence, it impaired HSC regenerative and self-renewing abilities in a cell-autonomous fashion. We revealed that Grb2 deletion impaired LT-HSC proliferative response to 5-FU treatment *in vivo*.

## Conclusions

Our findings position Grb2 as a key adaptor protein that governs cytokines signaling in cycling LT-HSC and as a master regulator of LT-HSC transplantation, proliferation, and survival.

# Abstract: Distribution of Corin and PCSK6 in Cardiac and Kidney Tissue in Experimental Model of Cardio-renal Syndrom. (Poster #73)

**Published:** 2018-01

**Authors:** Emad Khoury, Safa Kinaneh, Doron Aronson, Zaid Abassi

**Affiliations:** 1 Department of Physiology, The Ruth & Bruce Rappaport Faculty of Medicine, Technion–Israel Institute of Technology, Haifa, Israel; 2 Department of Cardiology, Rambam Health Care Campus, Haifa, Israel; 3 Biomedical Laboratory, Rambam Health Care Campus, Haifa, Israel; 4 The Ruth & Bruce Rappaport Faculty of Medicine, Technion–Israel Institute of Technology, Haifa, Israel

## Background

Corin and PCSK6 are key enzymes in the activation of natriuretic peptides, and play a vital role in the pathogenesis of congestive heart failure (CHF). The alterations in corin and PCSK6 expression have not been studied simultaneously in the cardiac and renal tissues in cardio-renal syndrome (CRS).

## Study Aims

To examine the status of PCSK6 and corin in the cardiac and renal tissues, 1 and 2 weeks from the induction of CHF.

## Materials & Methods

Congestive heart failure was induced in Sprague-Dawley rats by surgical formation of an aorto-caval fistula (ACF) (*n*=5–10). Sham-operated rats served as controls (*n*=5–10). Urinary Na+ excretion (UNaV) was determined in 1 or 2 weeks’ periods, after which the animals were sacrificed and their hearts, kidneys, and lungs were harvested. Corin and PCSK6 gene and protein expression levels were assessed by qPCR, western blotting, and immunofluorescence methods.

## Results

As expected, UNaV was lower in CHF rats as compared with sham-operated animals. Heart/ body weight ratio, an index of cardiac hypertrophy, increased by 36% and 45% (*P*<0.01) 1 and 2 weeks after ACF surgery, respectively. Similarly, lung/body weight ratio, an index of lung edema, increased by 51% and 38% (*P*<0.05) in rats with ACF after 1 and 2 weeks, respectively. In contrast, kidney/body weight decreased in rats with CHF 28% and 27% at 1 and 2 weeks after CHF induction, respectively. Corin mRNA and immunoreactive protein were detected in all heart chambers, where immunohistochemistry analysis revealed its localization at the myocyte membrane and in the apical membrane of proximal tubule, the medullary thick ascending limb of Henle’s loop (mTAL), and the collecting duct (CD) in the renal tissue. The expression levels of corin in cardiac chambers, namely right atrium, left atrium, right ventricle, and left ventricle, as well as in the renal tissue, significantly increased 1 week after CHF induction by 51%, 96%, 171%, 74%, and 269% (*P*<0.05), respectively. In contrast, corin abundance declined after 2 weeks, both in the cardiac and renal tissues, by 64%–88% and 53% (*P*<0.05), respectively. Noteworthily, while the expression dynamics of cardiac PCSK6 followed a pattern similar to corin, renal PCSK6 expression remained enhanced throughout the follow-up period.

## Conclusions

These results suggest that locally produced natriuretic peptide in the nephron may act as an autocrine mediator of salt–water homeostasis. Moreover, we presume that the initial upregulation of cardiac and renal PCSK6/corin in rats with CHF may be responsible for atrial natriuretic peptide (ANP)/brain natriuretic peptide (BNP) overproduction characterizing this clinical setting. The decline in corin as the disease progresses may contribute to the blunted natriuretic response to these peptides in heart failure.

# Abstract: Applying MDCLone, an Electronic Medical Record Search Tool, to Detect Prescription Errors in a Tertiary Care Hospital. (Poster #60)

**Published:** 2018-01

**Authors:** Laila Nassar, Yael Lurie, Daniel Kurnik

**Affiliations:** 1 Clinical Pharmacology and Toxicology Section, Rambam Health Care Campus, Haifa, Israel; 2 The Ruth & Bruce Rappaport Faculty of Medicine, Technion–Israel Institute of Technology, Haifa, Israel

## Background

Prescription errors are common and result in significant financial and health burdens. Rambam Medical Center’s electronic medical record (Prometheus) has no integrated computerized decision support system (CDSS) for prescription error alerting, and there is no information on the incidence of prescription errors.

## Study Aims

Our aim was to examine the feasibility of detecting selected prescription errors using MDClone (Beer Sheba, Israel), an electronic medical record search tool.

## Materials & Methods

We used MDClone to identify retrospectively three exemplary and clinically important prescription errors in Prometheus:

1. Omission of proton pump inhibitors (PPI) prescription in patients receiving an oral anticoagulant and at least one antiplatelet agent (2007–2017).
2. Oral low-dose methotrexate given more frequently than once every week (2010–2017).
3. Lack of therapeutic drug monitoring (TDM) in patients receiving vancomycin for at least 4 days (2009–2017).

## Results

*Between 2007 and 2017*, 4,425 patients received an oral anticoagulant and at least one antiplatelet agent. Only 53% received gastric protection with PPI. The same percentage was observed in patients older than 65.

*Between 2010 and 2017*, 943 patients received a prescription for low-dose methotrexate intended for once weekly administration. Ten percent received methotrexate on ≥2 days during their first week, and 35% were discharged with recommendation of daily methotrexate instead of weekly.

*Between 2009 and 2017*, 5,383 patients were treated with vancomycin for at least 4 days, and only 46% had appropriate TDM. Even among patients with acute kidney injury, only 64% had TDM.

## Conclusions

Prescription errors are common, and MDClone is an effective tool for retrospectively assessing their incidence. Our results will inform the development and implementation of an effective prescription error CDSS.

# Abstract: Safety and Efficacy of Endoscopist-directed Balanced Propofol Sedation during Endoscopic Retrograde Cholangio-pancreatography. (Poster #54)

**Published:** 2018-01

**Authors:** Alon Lapidus, Ian Gralnek, Alain Suissa, Amir Klien, Kamel Yassin, Iyad Khamaysi

**Affiliations:** 1 The Ruth & Bruce Rappaport Faculty of Medicine, Technion–Israel Institute of Technology, Haifa, Israel; 2 Department of Gastroenterology, Rambam Health Care Campus, Haifa, Israel

## Background

Endoscopist-directed balanced propofol sedation (BPS), defined as a fixed dose of an opioid and benzodiazepine combined with incremental doses of propofol, has been shown to be a safe and effective moderate sedation regimen for routine endoscopy. However, there are very limited data on the use of BPS in endoscopic retrograde cholangio-pancreatography (ERCP).

## Study Aims

The aim of this study was to evaluate the safety and efficacy of endoscopist-directed BPS, as well as to compare patient outcomes with anesthesiologist-administered moderate sedation, for both in-patients and ambulatory ERCP.

## Materials & Methods

We performed a retrospective cohort study using prospectively collected endoscopy data from a tertiary care, university-affiliated medical center where endoscopist-directed BPS during ERCP is routine practice. Patient-level demographics and pre-/post-procedure vital signs were collected along with BPS drug dosages, American Society of Anesthesiologists (ASA) score, and measured “hard endpoint” patient outcomes, including: patient need for bag-mask ventilation or endotracheal intubation, aborted ERCP due to sedation effects, need for hospital admission post-ERCP (out-patients only) or need for a change in the level of hospital care (in-patients only), and mortality within 24 hours of ERCP.

## Results

From October 2015 to March 2017, 501 patients underwent ERCP and received endoscopist-directed BPS (Cohort 1: 380 [76%] in-patient, mean age 64.1 years, 46% males, 24% ASA I, 65% ASA II, 11% ASA III). During this same time period, 24 patients received anesthesiologist-administered moderate sedation (Cohort 2: 19 [79%] in-patient, mean age 65.0 years, 67% males, 12% ASA I, 25% ASA II, 38% ASA III, 25% ASA IV). BPS dosages (mean±SD, range) were: fentanyl 0.06±0.02 mg, range 0.05–0.10 mg; midazolam 1.7±0.7 mg, range 1.0–2.5 mg; and propofol 178±103 mg, range 10–640 mg. The propofol dose was inversely correlated with patient age (*r*=−0.42, *P*<0.001), ASA score (*r*=−0.19, *P*<0.001), and Mallampati score (*r*=−0.24, *P*<0.001). No clinically meaningful differences were found in patient vital signs measured pre- and post-ERCP. Moreover, for Cohort 1, no patients required bag-mask ventilation, endotracheal intubation, nor hospital admission/change in level of in-hospital care following ERCP. One patient in Cohort 2 who received anesthesiologist-directed BPS required bag-mask ventilation, and the ERCP was aborted due to sedation effects. There was no mortality from any cause within 24 hours of ERCP. All patients were discharged from the advanced endoscopy suite without adverse events.

## Conclusions

Endoscopist-directed BPS appears feasible, safe, and efficacious for ASA I–III patients undergoing in-patient or ambulatory ERCP.

# Abstract: Interpersonal Functioning of Individuals Who Seek Bariatric Surgery Compared to a Control Group. (Poster #11)

**Published:** 2018-01

**Authors:** Yael Latzer, Inbal Globus, Anat Brunstein-Klomek

**Affiliations:** 1 Eating Disorders Institute, Division of Psychiatry, Rambam Health Care Campus, Haifa, Israel; 2 Faculty of Social Welfare and Health Sciences, The University of Haifa, Haifa, Israel; 3 School of Public Health, The University of Haifa, Haifa, Israel; 4 Interdisciplinary Center (IDC), School of Psychology, Herzliya, Israel

## Background

Studies have found that patients with binge eating disorder (BED) have difficulties in their interpersonal functioning. There is limited research examining interpersonal relationships among bariatric patients. Interpersonal functioning refers to a wide variety of different and complementary abilities, including assertiveness, the capacity to initiate and maintain a conversation, the adequate expression of one’s own feelings, the ability for emotional regulation with one another, and more.

## Study Aims

The aim of this study was to assess the differences in interpersonal functioning between bariatric candidates and a control group including individuals with normal-weight, overweight, and obesity.

## Materials & Methods

A total of 166 adults seeking bariatric surgery in Israel (mean BMI 40.7, SD 5.1), including 52 men and 114 women (mean age 43.2, SD 11.3), participated in the study. They were compared to 82 control participants who were divided into three groups: normal weight (*n*=41) (mean BMI 22.1, SD 1.8), including 9 men and 32 women (mean age 41.4, SD 9.1); overweight (*n*=27) (mean BMI 26.9, SD 1.4), including 15 men and 12 women (mean age 41.8, SD 8.03); and obese (*n*=14) (mean BMI 34.05, SD 4.05), including 3 men and 11 women (mean age 52.7, SD 9.8). All participants completed surveys addressing demographics, eating disorders (EDE-Q), depression, anxiety, stress (DASS), and interpersonal functioning (SAS-SR, IIP).

## Results

Bariatric candidates had significantly more difficulties in “primary relationship” and were higher in being “too caring”. Eating and shape concerns, as well as anxiety levels, were significantly higher in the bariatric candidate group compared to two of the control groups (normal weight and overweight). Weight concern levels were also found to be significantly higher amongst bariatric candidates compared to all three of the sub-control groups.

## Conclusions

Our findings are in line with previous studies which found difficulties with intimate relationships and nurturance among patients with BED. The ability to communicate with each other, independence vs. interdependence, and intimacy are key aspects of intimate relationships. Having difficulties related to nurturance (being “too caring”) may refer to the tendency to put other people’s needs before your own. These findings may be part of the explanation why some people gain more weight than others, and may contribute to understanding more about the failures of bariatric surgery.

# Abstract: Hyperbaric Treatment for Hydrogen Peroxide Poisoning Associated with Portal Venous Gas and Neurological Symptoms: A Case Report. (Poster #43)

**Published:** 2018-01

**Authors:** Barak Levit, Yael Lurie, Igor Markovich, Yoram Kluger, Anna Bloom Krasik, Ronen Bar, Yedidia Bentur

**Affiliations:** 1 Department of General Surgery, Rambam Health Care Campus, Haifa, Israel; 2 The Ruth & Bruce Rappaport Faculty of Medicine, Technion–Israel Institute of Technology, Haifa, Israel; 3 Department of Clinical Pharmacology and Toxicology, Rambam Health Care Campus, Haifa, Israel; 4 Israel Naval Medical Institute, Israel Defense Forces, Haifa, Israel

## Background

Hydrogen peroxide (H_2_O_2_) is an oxidizing agent. The strength of the oxidizing reaction is determined by the concentration.

The release of oxygen may cause gastrointestinal distension, as well as venous or arterial gas embolization. Ingestion of a small amount of 3% H_2_O_2_ can cause gastrointestinal irritation; concentrated (>10%) solutions can cause caustic injury and gas embolization. The volume of oxygen liberated from the decomposition of 30 mL of 35% H_2_O_2_ solution is 3.5 L.

We present a case of portal vein gas embolism due to concentrated H_2_O_2_ ingestion, successfully treated by hyperbaric oxygen.

## Case Report

A 47-year-old female presented to our emergency department (ED) after unintentional ingestion of a mouthful of 30% H_2_O_2_ solution used for swimming pool cleaning. She had a large self-induced frothy non-bloody vomiting episode at home.

On admission her pulse was 67 beats per minute, blood pressure 157/86 mmHg, and oxygen saturation 94% (room air). She complained of headache, abdominal pain, dizziness, and upper limb weakness. A thorough neurological examination revealed no objective findings. Abdominal computerized tomography (CT) scan showed multiple portal vein gas emboli. Electrocardiogram (ECG), biochemistry and hematological laboratory workup, laryngoscopy, brain CT, and cardiac echocardiography were normal.

She was treated in a hyperbaric chamber according to US Navy Diving Manual Treatment Table 6 (4.75 hours of oxygen and air intermittently up to 18 m depth) 8 hours after ingestion, with resolution of symptoms.

Post-treatment upper gastrointestinal endoscopy revealed diffuse gastritis. Follow-up abdominal CT performed 8 hours after completion of hyperbaric treatment showed complete disappearance of the gas emboli.

## Discussion

Concentrated H_2_O_2_ ingestion is an uncommon poisoning. It can cause severe complications, including hemorrhagic gastritis, respiratory collapse, cerebral gas embolism, pneumomediastinum, and death. Clinical manifestations are usually evident shortly after ingestion, but they may be delayed and abrupt.

Abdominal CT is a sensitive measure to detect portal gas emboli. Although a favorable outcome was reported even with delayed hyperbaric treatment, permanent disability has been recorded.

Early hyperbaric treatment can cause rapid resolution of gas emboli and may prevent neurologic complications.

We recommend performing abdominal CT in patients ingesting concentrated H_2_O_2_ and to consider early hyperbaric treatment in the presence of portal or systemic gas emboli, even if patients are asymptomatic.

# Abstract: Physical Activity in Type 1 Diabetes: Education and Barriers. (Poster #15)

**Published:** 2018-01

**Authors:** Ruth Livny, Wasim Said, Smadar Shilo, Nehama Zuckerman-Levin, Shoshana Gal, Naim Shehadeh, Michal Cohen

**Affiliations:** 1 Department of Pediatrics, Rambam Health Care Campus, Haifa, Israel; 2 Diabetes, Endocrinology, and Metabolism Institute, Rambam Health Care Campus, Haifa, Israel; 3 The Ruth & Bruce Rappaport Faculty of Medicine, Technion–Israel Institute of Technology, Haifa, Israel

## Background

The advantages of physical activity are particularly emphasized in children with type 1 diabetes, and 60 minutes of regular daily activity is recommended. The safe performance of activity requires diabetes-specific exercise education, and this is provided during clinic visits. Yet, reports suggest that children with type 1 diabetes perform less than the recommended daily activity.

## Study Aims

The aims of this study were: (1) To identify barriers and sources of support for exercise performance in children and adolescents with type 1 diabetes; and (2) to evaluate the education received and patients’ knowledge regarding exercise performance.

## Materials & Methods

Patients with type 1 diabetes 2–20 years of age, followed at the pediatric diabetes clinic of Rambam Medical Center, were recruited while attending a routine visit. Patients (over 18 years of age) or parents of patients under the age of 18 signed an informed consent. They then completed a questionnaire assessing demographic and health data, physical activity and barriers to its performance, family and social support, well-being, and diabetes-related exercise education.

## Results

A total of 102 patients with type 1 diabetes were included in this study. Mean age was 13.2±4.2 years; 53 were females. Median weekly time of reported physical activity was 4 hours (range 0–22), and only 28 participants (27%) were physically active at least 7 hours weekly. The three most prevalent perceived barriers to exercise were risk of hypoglycemia, low fitness, and weather (reported by 75%, 51%, and 47% of patients, respectively). Family support scores were generally favorable apart from those variables reflecting active exercise participation for which scores were in the lower half for over 50% of participants. On the other hand, social support scores were the highest for exercising together, and this variable correlated with the amount of activity performed (*r*=0.360, *P*<0.001). The majority of patients (97%) reported that the importance of physical activity was discussed with them in clinic, and 97% of them reported satisfaction from the clinic's exercise teaching. Seventy-five percent reported making adjustments in either food, insulin, or activity in order to control glucose during activity, yet only 42% of participants responded correctly to a set of questions regarding the glucose-lowering effect of exercise.

## Conclusions

We found that children with type 1 diabetes mellitus are performing less than the recommended daily physical activity. Hypoglycemia is perceived to be a major barrier, and increased social support may be an important factor to encourage activity. Increased efforts are required in order to increase knowledge and the amount of safely performed activity.

# Abstract: A Novel Homozygous Truncating Mutation in *SPTBN4* Underlies a Lethal Form of Atypical Charcot–Marie–Tooth Disease with Central Nervous System Involvement. (Poster #52)

**Published:** 2018-01

**Authors:** Daniella Magen, Ayala Ofir, Ajami-Yousef Maha, Ayelet Eran, Adi Mory, Hanna Mandel

**Affiliations:** 1 Pediatric Nephrology Institute, Ruth Rappaport Children’s Hospital, Rambam Health Care Campus, Haifa, Israel; 2 The Ruth & Bruce Rappaport Faculty of Medicine, Technion–Israel Institute of Technology, Haifa, Israel; 3 Laboratory of Molecular Medicine, Rambam Health Care Campus, Haifa, Israel; 4 Clalit Health Services, Kabul, Israel; 5 Department of Clinical Imaging, Rambam Health Care Campus, Haifa, Israel; 6 The Genetics Institute, Rambam Health Care Campus, Haifa, Israel; 7 Metabolic Unit, Rambam Health Care Campus, Haifa, Israel

## Background

Charcot–Marie–Tooth disease (CMTD) is a heterogeneous group of hereditary peripheral sensory and motor neuropathies, causing progressive distal limb muscle weakness, skeletal deformities, and decreased tendon reflexes. To date, more than 80 genes have been identified in association with CMTD. Causative genes are involved in neuronal and myelin development, axonal transport, and ion-channel function. β-IV spectrin, encoded by

*SPTBN4*, is a non-erythrocyte β-spectrin protein, regulating sodium channel clustering at the axonal initial segments and nodes of Ranvier. In mice, *Sptbn4* disruption has been shown to underlie a progressive auditory and motor neuropathy. Recently, a loss-of-function mutation in *SPTBN4* has been identified in a single Kurdish patient with congenital neuropathic myopathy and central deafness. So far, no additional human subjects with *SPTBN4* disruption have been reported.

## Study Aims

The aim of this study was to investigate the clinical features and the molecular basis underlying a lethal form of atypical CMTD, in a highly consanguineous Israeli Arab-Moslem family.

## Materials & Methods

Six patients with an autosomal-recessive form of atypical CMTD, and 12 of their close relatives, were included in the study. Phenotypic evaluation of affected individuals included extensive biochemical and metabolic laboratory work-up, muscle biopsies, electrophysiological studies, and brain MRI. Genomic DNA analysis included homozygosity mapping and whole-exome sequencing. RNA and protein expression analyses were performed on brain tissue from an aborted affected fetus.

## Results

Patients exhibited progressive limb motor and sensory neuropathy, psychomotor regression, cranial nerve defects, and central deafness. Death occurred within the first two decades, mainly from aspiration pneumonia. Extensive metabolic investigation was unremarkable. Muscle biopsies revealed fiber grouping, suggesting chronic denervation. Auditory brain stem evoked potentials revealed central deafness. Electroretinogram and visual evoked potentials indicated cone and rod dystrophy. Brain MRI demonstrated progressive cerebral and cerebellar atrophy. Exome sequencing revealed a homozygous deletion in exon 12 of *SPTBN4* (c.1453delG), predicted to cause frame shift and premature termination at residue 520 (p.Val485Cys fs*520), out of 2,564 residues in the expected full protein sequence. The c.1453delG mutation segregated with the disease phenotype in all study participants. *SPTBN4* expression analysis in brain tissue of an affected fetus revealed no RNA or protein expression, compatible with mRNA decay and complete protein loss-of-function.

## Conclusions

Our finding reinforces the role of *SPTBN4* in the pathogenesis of atypical CMTD, and enables genetic counseling, prenatal diagnosis, and high-risk population screening. The molecular mechanisms whereby *SPTBN4* loss-of-function results in the disease phenotype remain to be delineated.

# Abstract: The Interplay between Mitochondrial Dysfunction, Dopamine, and Hypomyelination: Implications for Schizophrenia. (Poster #34)

**Published:** 2018-01

**Authors:** Joumana Milhem, Ofer Yitzhaki, Rachel Karry, Dorit Ben-Shachar

**Affiliations:** 1 Research Laboratory of Psychobiology, Department of Psychiatry, Rambam Health Care Campus, Haifa, Israel; 2 The Ruth & Bruce Rappaport Faculty of Medicine, Technion–Israel Institute of Technology, Haifa, Israel

## Background

Schizophrenia (SZ) is one of the most disabling brain disorders. Abnormalities in dopaminergic transmission and white matter have been documented in SZ patients. An event causality between dopamine increase and hypomyelination in rat brain has been suggested. However, there is little information available on the relationship between these two anomalies. Mitochondrial dysfunction has been repeatedly reported in the disease, and their interaction with dopamine as well as toxic effects on myelin was demonstrated.

## Study Aims

The aim of this study is to investigate the interaction between dopamine, mitochondria, and myelin.

## Materials & Methods

Oligodendrocyte (OLN93) cells were grown in high-glucose Dulbecco's modified Eagle's medium (DMEM) supplemented with 2 mM L-glutamine, 100 U/mL streptomycin/penicillin, and with either 10% fetal calf serum (FCS), or 0.5% FCS+10 nM biotin, or a combination of growth factors. Cells were treated with different drugs: dopamine (50 μM), the mitochondrial complex I inhibitor rotenone (50 nM), or cuprizone (100 μM), a copper chelator causing demyelination and mitochondrial toxicity. Myelin basic protein (MBP) and proteolipid protein (PLP), major myelin proteins, were assessed by immunocytochemistry. Oxygen utilization rate in intact cells was measured polarographically. Mitochondrial network dynamics and membrane potential (ΔΨm) were measured by confocal microscopy following cell staining with the mitotracker dye JC-1.

## Results

Differentiation stage of cells, determined by MBP and PLP, depends on the media content. Cells grown with 10% FCS stay at their precursor stage, those transferred to biotin+0.5% FCS express significantly more MBP than the precursors, suggesting their differentiation, while cells transferred to medium containing various growth factors express significantly higher levels of MBP as well as PLP. One-way ANOVA revealed significant differences in MBP between the various drug treatments at all differentiation stages (*P*<0.001), while PLP was not affected by any of the drugs. Dopamine highest effect was detected in differentiated oligodendrocytes, while rotenone and cuprizone demonstrated a biphasic effect on MBP expression along differentiation. In addition, average basal oxygen consumption rate was 1.152±0.09 μmol/h/5×10^−6^ cells. Rotenone and cuprizone reduced respiration in all stages, while dopamine increased respiration in precursor cells, but inhibited it in differentiated oligodendrocytes. Preliminary data show that ΔΨm was also reduced by rotenone in differentiated cells (0.74±0.07 vs. 1.05±0.1 red/green fluorescence intensity ratios, *P*<0.025).

## Conclusions

Our preliminary data suggest that different treatments affecting mitochondria, such as dopamine, rotenone, and cuprizone, alter the major protein content of myelin in oligodendrocytes, depending on cell differentiation stage. This may suggest mitochondria as a link between myelin and dopamine.

# Abstract: Research-based Exome Sequencing to Provide a Molecular Diagnosis for Patients with Epilepsy. (Poster #51)

**Published:** 2018-01

**Authors:** Adi Mory, Nina Ekhilevitch, Tova Hershkovitz, Maya Steinberg, Alina Kurolap, Tamar Paperna, Aharon Schif, Sarit Ravid, Hagit N. Baris

**Affiliations:** 1 The Genetics Institute, Rambam Health Care Campus, Haifa, Israel; 2 The Ruth & Bruce Rappaport Faculty of Medicine, Technion–Israel Institute of Technology, Haifa, Israel; 3 Pediatric Neurology Unit, Ruth Rappaport Children’s Hospital, Rambam Health Care Campus, Haifa, Israel

## Background

Seizures are a common feature in numerous Mendelian and neurodevelopmental disorders, including autism spectrum disorder and intellectual disability. Several recent studies have highlighted the usefulness of whole-exome sequencing (WES) in a clinical pediatric neurology practice, emphasizing a high diagnostic rate and impact on genetic counseling and patient management.

## Study Aims

The aim of this study was to study the genetic basis of epileptic disorders.

## Materials & Methods

The Genetics Institute at Rambam Health Care Campus has formed a collaboration with the Department of Human Genetics, Yokohama City University Graduate School of Medicine, Japan, to study the genetic basis of epileptic disorders. Throughout 2015–2017, 44 families with at least one child with either syndromic or non-syndromic seizures were phenotyped in the genetics clinic and recruited to the project. Trio-based WES was utilized, and results were reported if pathogenic, likely-pathogenic, or no genetic cause found. Findings unrelated to the seizure phenotype or of unknown clinical significance were not reported.

## Results

To date, of 44 recruited families, 33 had reported results. Pathogenic or likely-pathogenic variants were found in 15 of 33 families (45%), two of which were identified to have candidate variants in novel genes. Of the 13 families with findings in known seizure-related genes, autosomal dominant inheritance was most commonly observed (*n*=8, 61.5%), mostly *de novo*; one family had X-linked recessive inheritance (7.7%), and four families had autosomal recessive inheritance (30.8%). One of the families had two causative epilepsy genes, resulting in double diagnosis. Pathogenic variants were found in *NARS2*, *SYNGAP1*, *GNAO1*, *PIGA*, *GLRA1*, *TMEM165*, *DNMT1*, *BRAF*, *TUBA1A*, *PGAP1*, and *SCN1A.*

## Conclusions

Epilepsy, whether syndromic or non-syndromic, is characterized by genetic heterogeneity. This influences genetic testing by requiring multi-gene approaches, such as gene panels and WES. We present a cohort of patients with seizure-related disorders that were referred to the genetic Institute at Rambam. The findings yielded by genetic analysis are in line with the literature, including the prevalence of autosomal dominant inheritance found in both non-consanguineous and consanguineous families.

# Abstract: Treatment Beyond Disease Progression: Anaplastic Lymphoma Kinase (ALK) Inhibitors in ALK-rearranged Advanced Non-small Cell Lung Cancer. (Poster #21)

**Published:** 2018-01

**Authors:** Mor Moskovitz, Alexandra Pavel, Priscilla Matthews, Donyang Yang, Brendon Morganstein, Wei Xu, Penelope Bradbury, Geoffrey Liu, Frances Shepherd, Natasha Leighl

**Affiliations:** 1 Oncology Institute, Rambam Health Care Campus, Haifa, Israel; 2 Department of Medical Oncology and Hematology, Princess Margaret Cancer Centre, University of Toronto, Toronto, Ontario, Canada

## Background

Lung cancer is the leading cause of cancer-related deaths worldwide. A subset of 4%–7% of non-small cell lung cancer (NSCLC) tumors harbors anaplastic lymphoma kinase (ALK) gene rearrangements, most commonly in younger patients, patients with adenocarcinoma, and never-smokers. The ALK tyrosine kinase inhibitors (TKI) are superior to chemotherapy in this population, with better outcomes, better convenience, and less toxicity. With the development of resistance, some patients are treated beyond disease progression rather than switching to alternative therapy. Potential reasons for this include limited access to options other than chemotherapy, oligoprogression (e.g. only in the central nervous system), toxicity profile, and patient and provider preference. The true benefit of treating patients beyond progression is unknown.

## Study Aims

The aim of this study was to evaluate the quality of life and cost of treatment beyond disease progression in ALK-positive NSCLC.

## Materials & Methods

Data were collected from consecutive ALK-rearranged advanced NSCLC patients treated at the Princess Margaret Cancer Centre from September 2010 to May 2017. Data were abstracted from the electronic patient records. Symptom burden was assessed by the Edmonton Symptom Assessment Scale (ESAS) at outpatient clinic visits. Drug acquisition costs for treatment were calculated using costs from the Princess Margaret Cancer Centre Outpatient Pharmacy.

## Results

Forty-six patients with ALK-rearranged advanced NSCLC were identified. The mean treatment duration until progression was 51 weeks, (range 1.4–261), and 26 patients (57%) received treatment beyond progression with their initial ALK TKI and remained on therapy for a mean of 7 months (range 0.8–30.5).

Mean symptom burden increased during treatment past progression, but 20% of patients continuing their initial ALK TKI past progression did experience ongoing symptom improvement. Half of those treated beyond progression went on to receive palliative radiation at progression. The mean cost per patient of initial ALK TKI therapy was $181,078 Canadian dollars (CAD). The mean cost per patient of treatment past progression was $47,548 CAD, 15% of the total cost of initial ALK TKI therapy.

## Conclusions

Patients with stable or improved symptom burden from baseline were more likely to continue on treatment past progression, while those with increased symptom burden did not. While treatment with ALK TKIs beyond progression has shown good clinical outcomes and can defer more toxic treatments like chemotherapy, the benefit for patients remains unclear and costly.

# Abstract: Common Patient-reported Symptoms with Health Utility Scores in Cancer Outpatients: Real World Comparison. (Poster #20)

**Published:** 2018-01

**Authors:** Mor Moskovitz, Kevin Jao, M. Catherine Brown, Susie Su, Hiten Naik, Lawson Eng, Tian Wang, Yvonne Leung, Wei Xu, Madeline Li, Gerald Devins, Doris Howel, Geoffrey Liu

**Affiliations:** 1 Oncology Institute, Rambam Health Care Campus, Haifa, Israel; 2 Department of Medical Oncology and Hematology, Princess Margaret Cancer Centre, University of Toronto, Toronto, Ontario, Canada

## Background

One form of health-related quality of life (HRQoL) assessments useful in economic analyses is the health utility score (HUS). Tools for measuring HUS such as the EuroQol (EQ-5D) were originally standardized to health state preferences in healthy individuals. These preferences are then applied secondarily to cancer patients.

## Study Aims

To demonstrate clinical appropriateness of using HUS to represent HRQoL, we assessed the extent to which the presence and severity of common cancer symptoms affected HUS generated from EQ-5D in cancer patients.

## Materials & Methods

Adult cancer outpatients at Princess Margaret Cancer Centre were cross-sectionally surveyed using the EQ-5D-3L, the Edmonton Symptom Assessment System (ESAS), and clinico-demographic variables. The HUS were derived from the EQ-5D-3L. Spearman correlation coefficients (*R*) were used to correlate ESAS symptoms with HUS. Multivariable linear regression analyses with stepwise selection identified independent variables associated with HUS.

## Results

Of 764 patients across multiple malignancies, 27% were palliative at assessment. Significant correlations were found between each ESAS symptom score and HUS (*P*<0.0001 for each comparison; Spearman coefficients ranged from 0.204 to 0.416). Correlations were strongest for pain (*R*=0.416), fatigue (*R*=0.387), and depression (*R*=0.354). After variable selection and adjustment for clinical variables, depression, and pain, symptom scores remained highly associated with HUS (*P*<0.0001 each). Prediction ability of ESAS to HUS was 0.25. The prediction ability of HUS by three of the ESAS questions—pain, depression, and anxiety—is 0.24, demonstrating that the ESAS is driven mostly by those three questions. Correlation of EQ-5D question (q) 4 to ESAS pain symptom was high, as well as correlation of EQ-5D q5 to the combined depression and anxiety symptoms in ESAS (Spearman correlation 0.90). The mapped HUS with EQ-5D q4 and q5 replaced by ESAS pain, depression, and anxiety is highly correlated with the real HUS (Spearman correlation 0.83).

## Conclusions

The HUS are associated with all major cancer symptoms. The EQ-5D-3L-derived HUS have clinical utility and can be used to assess the quality of health-care interventions. On exploratory analysis, we cannot replace the EQ-5D with the ESAS questionnaire, although we can replace the two symptom questions of the EQ-5D (q4 and q5) with the ESAS with high predictability.

# Abstract: Protease-Activated Receptor 1 Activity Mediates Granulocyte Colony-Stimulating Factor Induced Hematopoietic Stem Cell Mobilization and Predicts Their Repopulation Potential. (Poster #29)

**Published:** 2018-01

**Authors:** Neta Nevo, Shiri Gur-Cohen, Orit Kollet, Tsila Zuckerman, Benjamin Brenner, Myriam Ben Arush, Tsvee Lapidot

**Affiliations:** 1 The Joan and Sanford I. Weill Division of Pediatric Hematology-Oncology and Bone Marrow Transplantation, Ruth Rappaport Children’s Hospital, Rambam Health Care Campus, Haifa, Israel; 2 Department of Immunology, Weizmann Institute of Science, Rehovot, Israel; 3 Division of Hematology and Bone Marrow Transplantation, Rambam Health Care Campus, Haifa, Israel

## Background

Hematopoietic stem and progenitor cell (HSPC) mobilization by repeated granulocyte colony-stimulating factor (GCSF) treatment is the most frequently used protocol for allogenic transplantations. Our study in mice showed that the coagulation system has a role in controlling HSPC retention and egress in the bone marrow (BM). In particular the thrombin receptor protease-activated receptor 1 (PAR1) was found to be 3.3-fold higher in GCSF-mobilized human CD34+ HSPC compared to steady state BM CD34+ HSPC.

## Study Aims

The aim of this study was to realize the functional role of PAR1 in GCSF-induced mobilization and assess its clinical importance.

## Materials & Methods

We used functional pre-clinical models, where human cord blood HSPC were transplanted to immune-deficient irradiated mice, and analyzed coagulation involvement in their mobilization. Next, we analyzed the importance of PAR1 expression in GCSF-induced mobilization among 20 human healthy bone marrow donors: mononuclear cells were isolated, stained, and analyzed by fluorescence-activated cell sorting before and after GCSF-induced mobilization.

## Results

Using a chimeric mouse model, we found that GCSF induced PAR1 upregulation on human BM CD34+/CD38− HSPC. Antagonism of PAR1 signaling inhibited mobilization and induced accumulation of endothelial protein C receptor (EPCR)^high^ long-term repopulating HSPC in the BM, with high adhesion and homing potential. In addition, thrombin induced EPCR shedding from human HSPC, leading to their rapid mobilization. Next, we analyzed the significant clinical importance of our findings. We found that surface PAR1 expression levels on circulating mononuclear cells before GCSF treatment positively correlate with higher yields of mobilized leukocytes after stimulation. In addition, the percentage of PAR1 expressing CD34+ HSPC in the blood and their absolute numbers before treatment positively correlated with peripheral blood leukocyte count and yield of CD34+ HSPC. Moreover, as PAR1 expression on CD34+ cells is higher after stimulation with GCSF, the cells migrate better toward stromal cell derived factor 1 (SDF-1). Importantly, we found accelerated neutrophil and platelet recovery in patients transplanted with mobilized cells expressing higher surface PAR1 levels. Consequently, poor mobilizer donors are characterized by low PAR1 expression on circulating CD34+ cells prior to GCSF treatment. In addition, we present a thrombophilic donor, carrying the MTHFR mutation expressing low PAR1 levels at baseline, with the outcome of inadequate mobilization following treatment with GCSF.

## Conclusions

Taken together, our study identifies PAR1 signaling as an essential regulator of GCSF-induced HSC mobilization. PAR1 expression levels can be exploited and serve as a positive predictive marker for the efficiency of human CD34+ HSC mobilization among healthy donors in clinical GCSF-induced mobilization.

# Abstract: The Routine Use of Ureterovesical Stent in Transplanted Kidney and Its Effect on Post-Transplant Urological and Infectious Complications. (Poster #44)

**Published:** 2018-01

**Authors:** Rawi Ramadan, Haneen Daoud, Marina Tchircov, Aaron Hoffman, Suheir Assady

**Affiliations:** 1 Department of Nephrology and Hypertension, Rambam Health Care Campus, Haifa, Israel; 2 The Ruth & Bruce Rappaport Faculty of Medicine, Technion–Israel Institute of Technology, Haifa, Israel; 3 Department of Vascular Surgery, Rambam Health Care Campus, Haifa, Israel

## Background

Kidney transplantation is considered the treatment of choice in end-stage kidney disease. However, the transplantation procedure is associated with complications, which may negatively affect both graft survival and patient morbidity.

## Study Aims

The aims of this study were to examine the impact of prophylactic routine use of ureterovesical double J stent (DJS) in transplanted kidney on post-transplant urological and infectious complications, and to determine the effect of such complications on graft function.

## Materials & Methods

A total of 150 kidney transplant recipients at Rambam Health Care Campus between 2000 and 2014 were included. Patients were divided into two groups. The first group included 68 patients who received a routine DJS during the transplantation procedure (G1-DJS). The second group was a cohort of 82 patients who did not receive a ureteral stent (G2-NS). The rates of urinary leak, ureteral stricture, urinary tract infection (UTI), bacterial resistance profile, and graft function at three and six months post-transplantation were compared between the two groups.

## Results

### Urological Complications

Urinary leak rate was significantly reduced in G1-DJS compared with G2-NS (0% vs. 18%). Ureteral stricture rates were not different between the two groups (5.9% vs. 6.1%).

### Infectious Complications

Ureteral stents were not associated with an increase in UTI rate during the follow-up period. However, in multivariate analysis, diabetes was found to be a risk factor for UTI among patients who received ureteral stent (*P*=0.001). No correlation was found between time to stent removal and UTI rate. A significant increase in carbapenem-resistant bacterial infections was found in G1-DJS compared with the G2-NS cohort (*P*=0.024).

### Graft Function

Urological complications were associated with lower estimated glomerular filtration rate (eGFR) compared with no such complications at three and six months after transplantation (mean eGFRs: 48 mL/min/1.73 m^2^ vs. 61 mL/min/ 1.73 m^2^; and 54 mL/min/1.73 m^2^ vs. 60 mL/min/ 1.73 m^2^, respectively). Stent placement and UTIs had no significant influence on patients’ eGFR.

## Conclusions

Routine ureteric stenting significantly reduced urinary leak, and had no impact on UTI rate, except in diabetic patients. Urological complications had a detrimental effect on early to medium-term graft function. Therefore, the routine use of ureteral DJS in kidney transplantation is advisable, yet it warrants further prospective research.

# Abstract: A New Method for Radiotracer Concentration Measurement using SPECT/CT Technology—. (Poster #14)*In Vivo*/*In Vitro* Validation

**Published:** 2018-01

**Authors:** Ilya Reizberg, Rachel Lugassi, Savina Raviv, John A. Kennedy, Alex Frenkel, Ora Israel, Zohar Keidar

**Affiliations:** 1 Department of Nuclear Medicine, Rambam Health Care Campus, Haifa, Israel; 2 The Ruth & Bruce Rappaport Faculty of Medicine, Technion–Israel Institute of Technology, Haifa, Israel

## Background

The standard uptake value (SUV) is a quantitation tool used routinely in positron emission tomography (PET) imaging to measure radioactivity concentration in a defined lesion. The introduction of an equivalent index in single-photon emission computed tomography (SPECT) can improve the diagnostic accuracy of the imaging technique and improve the understanding of the investigated disease. New software that allows the absolute measurement of radiotracers in SPECT/CT studies has recently been developed and approved for clinical use (Q.Metrix software, GE HealthCare).

## Study Aims

The aim of this study is to estimate the accuracy of measuring the true concentration of an imaging radiotracer (Tc-99m methylene diphosphonate [MDP]) with SPECT/CT technology by means of urine sampling.

## Materials & Methods

Twenty patients referred for bone scintigraphy as part of their clinical assessment were included in a reference-controlled study (*in vivo*/*in vitro*).

After bone scintigraphy had been performed (according to the standard protocol and after injecting 925 MBq of Tc-99m MDP), including SPECT/CT of the pelvic area, urine samples were taken from each patient. The absolute concentration of the radiotracer (Bq/mL) in the bladder was measured by the application for SPECT/CT (*in vivo*). Urine samples from each patient were obtained, and the decay-corrected radiotracer concentration was assessed by means of a well counter (*in vitro*).

A linear regression analysis comparing *in vivo* and *in vitro* measurements was performed for the Q.Metrix radiotracer concentration results and the radiotracer concentration from the urine samples. *P*<0.01 was considered as statistically significant.

## Results

Data of 20 patients were included in the study. A total of 20 pairs of quantitative SPECT/CT studies and urinary samples were available for further analysis. A significant correlation (*r*=0.97, *P*<0.001) was obtained between the *in vivo* and *in vitro* measured bladder.

## Conclusions

Urine radioactivity concentration can be reliably measured *in vivo* by SPECT/CT. The availability of non-invasive, non-laborious *in vivo* quantitation opens the potential for important clinical applications with the goal of better differentiating disease from benign processes as well as for assessing response to treatment.

# Abstract: Door to Computed Tomography: Stalk the Stroke. (Poster #12)

**Published:** 2018-01

**Authors:** Mor Saban, Heli Patito, Salama Rabia, Darawsha Aziz

**Affiliations:** 1 The Cheryl Spencer Department of Nursing, the University of Haifa, Haifa, Israel; 2 Department of Emergency Medicine, Rambam Health Care Campus, Haifa, Israel

## Background

Delayed diagnosis in patients with acute ischemic stroke still represents a blind spot in the assessment of quality health-care indicators.

## Study Aims

To evaluate a fast-track program to reduce “door to computed tomography” (CT) time and thrombolytic therapy in patients with suspected stroke admitted to emergency department.

## Materials & Methods

A retrospective archive study was conducted in 2017 on admissions between 2015 and 2016. We collected clinical data on all stroke patients (*n*=577) admitted to the ED at Rambam Health Care Campus, and compared the adherence to clinical guidelines before (*n*=309) and after (*n*=268) the intervention program was implemented.

The program comprised five steps:

1. The ED nurse and neurologist receive an announcement of the imminent arrival of a stroke patient.
2. The staff wait for the patient in the shock room.
3. The neurologist performs a rapid assessment according to the National Institutes of Health Stroke Scale (NIHSS), and simultaneously the nurse assesses the vital signs and takes blood tests.
4. The nurse contacts the CT unit.
5. A rapid exit (less than a 15 minute visit) from the shock room.

## Results

After implementing the intervention program, the median time to CT was 31 minutes compared to 51 minutes (*P*<0.001); 109 patients (35.2%) received thrombolytic therapy compared to only 79 patients (29.4%) (*P*=0.04); 61 patients (56%) vs. 50 (63.2%) underwent reperfusion with intravenous (IV) recombinant tissue plasminogen activator (rt-PA) (*P*=0.003). Mechanical clot disruption was performed for 21 (19.2%) vs. 15 (18.9%) (*P*=0.12) patients post- compared to pre-intervention, respectively. An increase in activity volume was also observed in integrated approach cases. Intravenous rt-PA and mechanical clot disruption was performed in 27 patients (24.7%) in 2016 and in 14 (17.7%) in 2015 (*P*=0.02).

After implementing the intervention, more patients received a neurologist evaluation within 10 minutes (72.3%) compared to pre-intervention (56.6%) (*P*=0.04), and more patients stayed in the ED for less than 60 minutes (68.2% vs. 41.7%, respectively, *P*=0.001).

## Conclusions

When comparing post- to pre-intervention, less time lag (in minutes) was measured in post patients. Thus, attaching a case manager to perform a rapid assessment of patients with acute ischemic stroke reduced the time for patients to receive a CT. These findings have significant implications for the quality of care for patients with suspected stroke admitted to an ED.

These findings also encourage further such interventions to achieve better outcomes in the critical assessment of stroke patients in the ED.

# Abstract: Quantitative Ultrasonometry: An Alternative and Easy Method to Evaluate Bone Mineral Density in People Living with Human Immunodeficiency Virus. (Poster #28)

**Published:** 2018-01

**Authors:** Elana Segal, Gamal Hassoun, Carcom Maor, Eduardo Shahar

**Affiliations:** 1 Diabetes and Endocrinology Institute, Rambam Health Care Campus, Haifa, Israel; 2 Immunology Unit, Rambam Health Care Campus, Haifa, Israel

## Background

Human immunodeficiency virus (HIV) infection and antiretroviral therapy (ART) are associated with bone mineral loss and poor vitamin D status in HIV-infected people. Measuring dual-energy X-ray absorptiometry (DXA) is the gold standard method to evaluate the status of bone mineral density (BMD). However, it is not always readily available. An alternative and easy method is needed to evaluate BMD in those infected with HIV.

## Study Aims

The aim of this study was to evaluate portable quantitative ultrasonometry (QUS) as an alternative technique to provide information about bone density, bone strength, and the bone turnover markers in HIV-infected people.

## Materials & Methods

A total of 69 men took part (34 HIV-infected men were matched with 35 non-HIV-infected men of the same age) in the tests. Bone mineral density was assessed by the Achilles quantitative ultrasonometer (GE Lunar, Madison, WI, USA) at the calcaneal heel. The HIV status was recorded for all HIV-infected patients. Calcium-regulating hormones and bone turnover markers were assessed in all participants.

## Results

The mean age was 47.8±7.8 years and 49.1±6.00 years for the HIV-infected and non-infected population, respectively. The bone mineral density was reduced in HIV-infected patients. The median T score was −0.65 (−1.13; 0.5) in the HIV-infected men vs 0.3 (−0.5; 0.9) in non-infected men (*P*=0.026). Bone turnover markers were higher in the HIV-infected patients, procollagen type I N propeptide (P1NO) (ng/mL) was 48.0±14.3 vs 41.1±15.2 (*P*=0.057), and carboxy-terminal collagen crosslinks (CTX) (ng/mL) was 0.41±0.18 vs 0.29±0.11 (*P*=0.002). We did not find an association between the traditional risk factors of reduced bone mineral density in the HIV-infected participants.

## Conclusions

Quantitative ultrasonometry is safe, easy to use, radiation-free, and the QUS devices are portable. Hence, QUS could be used for easy screening of bone mineral density status in HIV patients.

# Abstract: Novel Interaction of Human Bestrophin1 and Mesencephalic Astrocyte-derived Neurotrophic Factor (MANF) in the Retinal Pigment Epithelium. (Poster #09)

**Published:** 2018-01

**Authors:** Elena Segal, Ronit Heinrich, Shadi Safuri, Ami Aronheim, Naim Shehadeh, Ido Perlman

**Affiliations:** 1 Department of Neonatology, Ruth Rappaport Children’s Hospital, Rambam Health Care Campus, Haifa, Israel; 2 Department of Physiology and Biophysics, The Ruth & Bruce Rappaport Faculty of Medicine, Technion–Israel Institute of Technology, Haifa, Israel; 3 The Rappaport Institute for Biomedical Research, Haifa, Israel; 4 Department of Ophthalmology, Rambam Health Care Campus, Haifa, Israel; 5 Department of Molecular Genetics, The Ruth & Bruce Rappaport Faculty of Medicine, Technion–Israel Institute of Technology, Haifa, Israel; 6 Department of Endo-crinology, Rambam Health Care Campus, Haifa, Israel

## Background

Accumulation of toxic lipofuscin in the retinal pigment epithelium (RPE) damages the photoreceptors in the macula, leading to macular degeneration. Specific mutations in the human bestrophin1 (Best1) gene causes an autosomal dominant macular dystrophy known as Best vitelliform macular dystrophy (BVMD), as well as other ocular phenotypes, collectively termed bestrophinopathies. Best1 expression is confined to the RPE, responsible for phagocytosis of photoreceptor outer segments (POS). Lipofuscin accumulation is a hallmark of BVMD, and we suggest that Best1 modulates POS phagocytosis by interacting with regulatory proteins.

## Study Aims

The aim of this study was to identify RPE proteins that interact with Best1, and to characterize the interaction.

## Materials & Methods

Human RPE cDNA library was screened with wild-type (wt) Best1 using the Ras recruitment system, a cytoplasmic-based yeast two-hybrid system. The identified interaction was validated by co-immunoprecipitation (co-IP) and Duolink proximity ligation assay (DPLA) kit, and tested for dependency upon three Best1 mutations, as well as chloride and calcium concentrations.

## Results

One of the clones identified in the screening was mesencephalic astrocyte-derived neurotrophic factor (MANF), a novel neurotrophic factor that is expressed and secreted in response to endoplasmic reticulum stress. MANF acts as an anti-apoptotic factor in various tissues, including the retina where it protects photoreceptors from degeneration. Lysates from HEK293 cells expressing wt Best1 and MANF were subjected to co-IP, and Best1 was detected in the precipitated immunocomplexes, confirming wt Best1–MANF interaction in mammalian cells. The effect of Best1 mutations upon Best1–MANF interaction was evaluated: Glu300Asp- and Arg218Ser-mutated Best1 associated with BVMD failed to interact with MANF, while Val86Met-mutated Best1, associated with autosomal dominant vitreoretinochoroidopathy (ADVIRC), exhibited weaker interaction compared to wt Best1. DPLA experiments with stable lines prepared from ARPE-19 (human RPE cell line), expressing either wt Best1 or mutated variants, confirmed the results obtained by co-IP. Known Best1 functions, as Ca^+2^-regulated Cl^−^ channel and regulator of intracellular Ca^+2^ homeostasis, were tested for effects on Best1–MANF interaction. Applying 500 μM 4,4′-diisothiocyano-2,2′-stilbenedisulfonic acid (DIDS), a non-specific Cl^−^ channel blocker, diminished the wt Best1–MANF interaction but did not disrupt the weak interaction of ADVIRC mutation. Addition of 100 μM BAPTA-AM, a permeable Ca^+2^ buffer, did not affect wt Best1–MANF interaction but eliminated the weak interaction of ADVIRC mutation.

## Conclusions

We described a novel interaction: Best1–MANF in RPE cells. This interaction depends upon Best1 mutations and the Cl^−^ channel property of Best1, but not upon intracellular Ca^+2^ levels.

# Abstract: Pre-implantation Genetic Diagnosis Services for the Population of Northern Israel: A Summary of the First Years of the Reopened Rambam Pre-implantation Genetic Diagnosis Unit. (Poster #67)

**Published:** 2018-01

**Authors:** Hanna Segev, Tamar Paperna, Vered Ofen Glassner, Nivin Moustafa-Hawash, Nissreen Kinaani Mousa, Elena Milner, Shlomit Aharony, Ofer Fainaru, Shahar Kol, Hagit N. Baris

**Affiliations:** 1 The Genetics Institute, Rambam Health Care Campus, Haifa, Israel; 2 The In Vitro Fertilization Unit, Rambam Health Care Campus, Haifa, Israel; 3 The Ruth & Bruce Rappaport Faculty of Medicine, Technion–Israel Institute of Technology, Haifa, Israel

## Background

In 2015, Rambam reopened its Pre-implantation Genetic Diagnosis (PGD) Unit—the only one serving Northern Israel. The unit addresses an important need for many families from this region affected with monogenic disorders and chromosomal abnormalities. Rambam’s PGD Unit is a collaboration between Rambam’s Genetics Institute and *In Vitro* Fertilization (IVF) Unit.

## Study Aims

The aim of this study is to present a summary of the activity of Rambam’s PGD Unit from 2015 to 2017.

## Materials & Methods

Couples at risk receive genetic counseling at the Genetics Institute. If eligible, a family-specific PGD test is scheduled in Rambam’s PGD Unit. Following test approval, the couple undergoes IVF at the IVF Unit, and diagnosis at the PGD Unit. Unaffected embryos are transferred according to established protocols.

## Results

Since February 2015, 109 families underwent PGD counseling at the PGD Unit, and 80 families opted to continue with PGD. A total of 56 indications included monogenic disorders, microdeletions, and balanced translocations. Until July 2017, 53 PGD cycles for 31 families were performed in our unit. Of the 206 analyzed embryos, 64 were diagnosed as healthy, of which 55 were transferred in 41 cycles. Ten singleton pregnancies were achieved (24.3%) with one missed abortion and two pregnancy terminations due to embryo abnormalities that were unrelated to the PGD (which was confirmed as correct).

Among the 23 indications for rare monogenic disorders, founder mutations that are unique to populations residing in Northern Israel constitute a major portion of our PGD services. However, we have also implemented PGD services for new genes and diagnoses that were identified as part of the Rambam Genetics Institute genomic research program. For example, our discovery of mutations in the *SLC6A9* gene as the cause for a new glycinopathy has led to the birth of an unaffected child within a year.

## Conclusions

Rambam’s PGD services are now available for families from Northern Israel. These services are part of the Rambam Genetics Institute’s “one stop shop” for rapid translation of genetic research discoveries to clinical genetic services. We have observed an increase in demand over the past two years for the PGD Unit’s diagnosis and genetic counseling services. The rapid assimilation reflects the previously unmet need in the region.

# Abstract: CHD4/NuRD Related Intellectual Disability—Defining a New Syndrome. (Poster #17)

**Published:** 2018-01

**Authors:** Karin Weiss

**Affiliations:** Genetics Institute, Rambam Health Care Campus, Haifa, Israel

## Background

The chromodomain-helicase-DNA-binding protein 4 (CHD4) is a core component of the nucleosome remodeling and deacetylase (NuRD) complex, which possesses both chromatin remodeling and histone deacetylation activities. Both CHD4 and NuRD are involved in stem cell differentiation, embryonic development, and oncogenesis. Somatic variants in *CHD4* were identified as driver mutations in serous endometrial carcinoma. More recently *de novo* germline variants in *CHD4* were found to cause a multiple congenital anomaly and intellectual disability syndrome (MIM# Sifrim–Hitz–Weiss syndrome). The individuals that were first described shared several features such as macrocephaly, hypogonadism, congenital heart anomalies, and certain dysmorphic features. In addition, most had missense substitutions affecting the ATPase/helicase domains of the protein, and a dominant negative mechanism has been proposed.

## Study Aims

The aim of this study was to better define the clinical spectrum of the disorder and identify genotype–phenotype correlations.

## Materials & Methods

Clinical information was collected from 23 subjects with *de novo* variants in *CHD4*. Consents were obtained for the publication of photographs. The participants underwent whole-exome sequencing for developmental delay and/or congenital anomalies at different institutes in Europe and North America.

## Results

The most common clinical findings were global developmental delay, intellectual disability, dysmorphic features, heart defects, hypogonadotrophic hypogonadism, macrocephaly, ventriculomegaly, hearing loss, bone fusions, and growth delay. Two individuals were diagnosed with congenital moyamoya disease which supports a link between CHD4 abnormalities and this rare condition. There were no reports of malignancies; however, the median age in the cohort was 10 years. Therefore, we cannot rule out an increased risk for malignancies during adulthood. The majority of participants had missense variants in the ATPase (SNF2-like) domains. Individuals with variants within the ATPase domain were more likely to have typical clinical features. Two participants with a non-specific presentation had truncating variants. Since truncating variants in CHD4 were seen in unaffected individuals, it is not clear whether those are causative or not.

## Conclusions

The investigation of a larger cohort of individuals with *de novo CHD4* variants identifies previously described characteristic clinical features and supports a link with moyamoya syndrome. However, the interpretation of non-syndromic cases and truncating variants is challenging. Further studies on the clinical presentation of CHD4-related intellectual disability and functional characterization of uncertain variants will help with patient management and family counseling.

# Abstract: Involvement of Nitric Oxide and Endothelin Systems in the Pathogenesis of Endothelial Dysfunction in Diabetic Nephropathy. (Poster #77)

**Published:** 2018-01

**Authors:** Hiba Yaseen, Niroz Abu-Saleh, Hoda Awad, Mogher Khamaisi, Zaid Abassi

**Affiliations:** 1 Department of Internal Medicine D, Rambam Health Care Campus, Haifa, Israel; 2 The Ruth & Bruce Rappaport Faculty of Medicine, Technion–Israel Institute of Technology, Haifa, Israel; 3 Department of Physiology, The Ruth & Bruce Rappaport Faculty of Medicine, Technion–Israel Institute of Technology, Haifa, Israel; 4 Department of Laboratory Medicine, Rambam Health Care Campus, Haifa, Israel

## Background

Endothelial dysfunction (ED) is a key feature of diabetes, and is a major cause of vascular complications characterizing diabetic nephropathy. Diabetic patients who also exhibit hyperlipidemia suffer from accelerated vascular complications. While the deleterious effects of high glucose levels (HG) and hyperlipidemia alone on ED are well known, the effects of combined hyperlipidemia and HG have not been thoroughly studied.

## Study Aims

The aim of this study was to investigate whether a combination of hyperglycemia and hyperlipidemia aggravates diabetes-associated endothelial dysfunction.

## Materials & Methods

We used a multidisciplinary approach including cultured macrovascular human umbilical vein endothelial cells (HUVECs), a human microvascular endothelial cell line (HMEC-1), and knockout mice C57BL/ 6JLDLr^tmlHer^ (LDLR^−/−^) to investigate the mechanisms underlying combined HG and hyperlipidemia-induced ED. Incremental doses of glucose in the presence or absence of oxidized LDL (oxLDL) were added to HUVECs. After 5 days, the status of nitric oxide (NO) and endothelin (ET) systems, as well as their signal transduction, were assessed using western blot, ELISA, and immunoreactive staining. The effects of chronic combination of HG and hyperlipidemia on endothelial integrity and function as well as alterations in nitric oxide and endothelin systems in vascular, cardiac, and renal tissues were examined in knockout mice LDLR^−/−^ and their wild type (BALB).

## Results

Both microvascular and macrovascular endothelial cells exposed to HG and oxLDL, or a combination of both, displayed enhanced ET-1 production. Overproduction of ET-1 stems from upregulation of endothelin converting enzyme (ECE-1) as observed under these conditions. In contrast, the combination of HG and oxLDL dramatically decreased both total endothelial NO synthase (eNOS) by 60%, and activated eNOS (peNOS) by 50%. Moreover, NRF2 (transcription factor for various antioxidant enzymes) decreased by 42% and its active form (pNRF2) by 56%, as compared to baseline. Likewise, endothelin receptor type B (ET_B_) levels on endothelial cells decreased by 64% from baseline. Diabetic LDLR^−/−^ mice had higher blood pressure compared with normoglycemic LDLR^−/−^ and BALB mice (135±2 vs. 113±4 and 97±4 mmHg, respectively), higher plasma glucose (308±52.7 vs. 159.2±18.8 and 105±10.51 mg%, respectively), higher ET-1 (14.9±0.9 vs. 12.31±2.097 and 9.57±0.497 pg/mL, respectively), and higher NO_2_/NO_3_ levels (42.3±21.71 vs. 12.65±1.24 and 12.76±2.17 μM, respectively).

## Conclusions

Combined diabetes and hyperlipidemia is characterized by activation of the ET system and attenuated eNOS and NRF2 level/activity. These findings suggest that perturbations in these paracrine systems may contribute to diabetic nephropathy.

# Abstract: Cyclosporine A induces Endothelin-Converting Enzyme (ECE)-1: Studies . (Poster #64)*In Vivo* and *In Vitro*

**Published:** 2018-01

**Authors:** Hiba Yaseen, Samuel N. Heyman, Zaid Abassi, Christian Rosenberger, Natalia Kits, Galia Skarjinski, Mogher Khamaisi

**Affiliations:** 1 Department of Medicine D, Rambam Health Care Campus, Haifa, Israel; 2 Clinical Research Institute at Rambam, Rambam Health Care Campus, Haifa, Israel; 3 Department of Medicine, Hadassah Hebrew University Hospital, Mt. Scopus, Jerusalem, Israel; 4 Department of Physiology, The Ruth & Bruce Rappaport Faculty of Medicine, Technion–Israel Institute of Technology, Haifa, Israel; 5 Department of Laboratory Medicine, Rambam Health Care Campus, Haifa, Israel; 6 Department of Nephrology, Charité, Berlin, Germany; 7 The Ruth & Bruce Rappaport Faculty of Medicine, Technion–Israel Institute of Technology, Haifa, Israel

## Background

Endothelin-converting enzyme (ECE)-1 plays a key role in the generation of endothelins (ET) from their precursors—big endothelins. Previously, we have reported that ECE-1 expression is triggered by hypoxia via hypoxia-inducible factors (HIF), and demonstrated enhanced ECE-1 induction predominantly in distal nephron segments in the diabetic kidney and following the administration of radiocontrast medium. Both conditions are characterized by intensified renal hypoxia and HIF stabilization. Cyclosporine A (CsA) increases plasma ET-1 and induces renal vasoconstriction and hypoxia, conceivably participating in cyclosporine nephrotoxicity.

## Study Aims

The aim of this study was to examine whether CsA increases endothelin-1 (ET-1) generation and renal vasoconstriction via a feed-forward loop of hypoxia-driven, HIF-mediated enhanced ECE-1 induction.

## Materials & Methods

### In vivo

Animals (rats or mice) were randomized to a cremophore- or a CsA-treated group. Cremophore or CsA 120 mg/kg was injected daily subcutaneously at the back of the neck for four consecutive days. Animals were sacrificed two hours after the fourth injection. Kidneys were subsequently separated to cortex, outer medulla, and papilla. In some experiments rats were injected intravenously with 60 mg/kg pimonidazole (PIM) to detect hypoxia. Kidneys were analyzed by immunehistochemistry for ECE-1 expression and by western blot for ECE-1 and HIF 1-α protein expression.

### In vitro

Human umbilical vein endothelial cells (HUVECs) and human proximal tubular cells (HK-2 cells) were cultured in six well plates for 3 days, reaching confluence (90%), then treated with CsA )10 μmol/L). In additional experiments, dose- and time-dependent responses to CsA were determined by incubating HUVEC with CsA (0, 0.01, 0.10, 1.00, 10.00, and 100.00 μmol/L) for 24 hours, and by HUVEC subjected to CsA (10 μmol/L) for 0, 6, 12, 24, and 48 hours. ET-1 secretion was measured by enzyme immunoassay.

Real-time PCR was performed to evaluate mRNA expressions of ET-1 and ECE-1 in both cells lines.

## Results

We found that CsA intensifies renal parenchymal ECE-1 expression in the rat kidney (western blot and immunostaining), particularly in distal nephron segments, along with renal hypoxia (pimonidazole adducts) and HIF expression (western blot), in line with our recent observations showing episodic hypoxia in mice. Furthermore, in cultured normoxic endothelial and tubular cells, CsA dose-dependently induced ECE-1 mRNA and protein expression.

## Conclusions

In summary, CsA induces ECE-1 via both hypoxic and non-hypoxic pathways and may contribute to increased ET-1 production, generating a feed-forward loop of renal parenchymal hypoxia and ET synthesis.

# Abstract: The Relationship between Pityriasis Lichenoides and Mycosis Fungoides: A Clinicopathological, Immunohistochemical and Molecular Study. (Poster #69)

**Published:** 2018-01

**Authors:** Hiba Zaaroura, Dvora Sahar, Tova Bick, Reuven Bergman

**Affiliations:** 1 Department of Dermatology, Rambam Health Care Campus, Haifa, Israel; 2 Department of Hematology, Rambam Health Care Campus, Haifa, Israel; 3 Department of Pathology, Rambam Health Care Campus, Haifa, Israel

## Background

Pityriasis lichenoides (PL) has traditionally been classified with benign papulosquamous disorders. Patients with PL who develop mycosis fungoides (MF) are often described in the literature. It is unclear whether patients with PL who develop MF originally had MF, or if PL evolves into MF similar to what may occur in other benign dermatoses, and whether there are indicators of MF progression.

## Study Aims

The aim of this study was to find clues to the progression of PL to MF using clinical, histopathological, immunohistochemical, and molecular approaches.

## Materials & Methods

The histopathological diagnoses of all cases with PL diagnosed at the Rambam Health Care Campus during January 2000–December 2013 were retrieved; the clinical files of all included PL patients were searched for cases ultimately diagnosed as MF. The histopathological slides of these PL and MF lesions were re-examined and immunostained for CD3, CD4, CD8, CD7, CD30, and Ki-67. In addition, DNA was extracted from fresh biopsies or retrieved from paraffin sections and studied for T cell receptor (TCR) gene rearrangements (GR). The slides of the remaining PL cases which did not progress into MF were also studied histopathologically, with an emphasis on exocytosis (epidermotropism), follicular involvement, and nuclear atypia in the intraepidermal lymphocytes. There were 58 new PL cases (follow-up: 3–16 years, average: 8.3).

## Results

Three (5.2%) of the 58 PL patients developed MF after 3–11 years of prolonged clinical course. Papules and small plaques characterized PL, and patches and larger plaques subsequent MF. Thirty-five of 41 (85%) followed-up non-MF-associated PL patients reported lasting complete remissions. Histopathologically, apoptotic keratinocytes disappeared mostly or completely in subsequent MF. The presence of epidermotropism, folliculotropism, and epidermal lymphocytic nuclear atypia in PL was not predictive of MF. CD8+ cells were the dominant intraepidermal lymphocytes in the three PL patients, but remained so in only one subsequent MF case. CD7+ lymphocytes decreased substantially in two MF patients, and lymphocytic nuclear atypia increased markedly in one. T-cell receptor gene rearrangement (TCR-GR) studies demonstrated clonal populations in one of two studied PL patients and in all three subsequent MF cases.

## Conclusions

A few cases of PL may evolve into MF. Prolonged clinical course, the appearance of patches and larger plaques, markedly increased lymphocytic nuclear atypia, marked diminution of apoptotic keratinocytes and CD7+ and CD8+ lymphocytes, and clonal TCR-GR may serve as clues.

# Abstract: Vascular Alterations Play a Role in the Pathogenesis of Medication-related Osteonecrosis of the Jaw. (Poster #66)

**Published:** 2018-01

**Authors:** Hadar Zigdon Giladi, Gal Cohen, Lana Eskander, Tal Tamari, Talia Cohen

**Affiliations:** 1 Department of Periodontology, Rambam Health Care Campus, Haifa, Israel; 2 Bone Research Laboratory, Rambam Health Care Campus, Haifa, Israel; 3 Preclinical Research Authority, Technion–Israel Institute of Technology, Haifa, Israel

## Background

Medication-related osteonecrosis of the jaw (MRONJ) is a serious adverse effect of bisphosphonate, receptor activator of nuclear factor-kappaB ligand (RANKL) antibody, and vascular endothelial growth factor (VEGF) antagonist therapy. The condition is defined as an area of exposed necrotic bone in the maxillofacial region of patients on these pharmacological agents, without any history of radiotherapy. Furthermore, the lesions do not heal within 8 weeks after identification. Pain, bone sequestration, tooth loss, intraoral and extraoral fistulae, and jaw fracture are usually associated with MRONJ. Although rare, MRONJ is more common in oncological patients with concurrent use of chemotherapeutic regimens and steroids. The pathogenesis of MRONJ is not fully understood, and a standardized treatment protocol is lacking. Owing to the importance of adequate blood supply to bone turnover and wound healing, we hypothesize that the pathogenesis of MRONJ may involve vascular alterations in adjacent tissue.

## Study Aims

The aim of this study was to establish a MRONJ model in rats and to analyze VEGF levels and functional vascularization in MRONJ lesions.

## Materials & Methods

Thirty Lewis rats were treated with subcutaneous injection of either zoledronic acid (ZA) 7.5 μg/kg; dexamethasone (Dex) 1 mg/kg; ZA+Dex; or saline; once a week, for 11 weeks. At three weeks, the first molars were extracted bilaterally. Eight weeks following extraction, fluorescent dye was perfused to demonstrate functional vessels and rats were sacrificed. Blood was collected for serum VEGF analysis, and the extraction sites were examined clinically and histologically. Comparison between the groups was performed using the Kruskal–Wallis test.

## Results

The MRONJ-like lesions were evident in 50% of the ZA+Dex rats and in 20% of ZA rats. None of the rats in saline and Dex-only groups showed signs of MRONJ. Histological characteristics of MRONJ included necrotic bone, ulcerated epithelium, and inflammatory infiltrate. Systemic uses of ZA, Dex, and ZA+Dex were associated with vascular alterations: serum VEGF was lower in the ZA+Dex and Dex groups (*P*<0.05), whereas tissue VEGF was reduced in ZA+Dex and ZA groups compared to saline (*P*<0.05). Additionally, functional blood vessels were reduced in ZA+Dex and Dex groups compared to the saline group (*P*<0.05).

## Conclusions

Zoledronic acid and Dex interfere with angiogenic processes that may contribute to MRONJ occurrence. However, since Dex alone reduced VEGF and vessel density but was not associated with MRONJ, we conclude that impaired vascularity is a contributing factor but not the main cause for MRONJ.
